# Cryo‐Inactivated Cancer Cells Derived Magnetic Micromotors for Tumor Immunotherapy

**DOI:** 10.1002/advs.202504986

**Published:** 2025-05-23

**Authors:** Qingfei Zhang, Gaizhen Kuang, Wenzhao Li, Yuanjin Zhao

**Affiliations:** ^1^ Department of General Surgery The First Affiliated Hospital of Wenzhou Medical University Wenzhou 325035 China; ^2^ Wenzhou Institute University of Chinese Academy of Sciences Wenzhou 325001 China; ^3^ Department of Rheumatology and Immunology Nanjing Drum Tower Hospital School of Biological Science and Medical Engineering Southeast University Nanjing 210096 China

**Keywords:** cancer cell, ferroptosis, immunotherapy, micromotor, vaccine

## Abstract

Immunotherapy represents a highly promising modality in cancer treatment, with substantial advancements in therapeutic strategies. The primary challenge lies in enhancing the efficacy of immunotherapy approaches. Here, novel cryo‐inactivated cancer cells (CICC) derived magnetic micromotors (CICC@FeMnP) are reported for tumor synergistic immunotherapy. Through the magnetic control, the CICC@FeMnP micromotors can on‐demand target and accumulate at the tumor site. The FeMnP can induce ferroptosis and then trigger immunogenic cell death of tumor cells. The CICC containing the whole cancer antigen can conduct vaccination effects. Together with the Mn^2+^‐mediated cGAS‐STING pathway to stimulate the immune response, substantial anti‐tumor immune effects can be achieved. Importantly, the CICC@FeMnP micromotors not only facilitate the establishment of a collaborative anti‐tumor immune network to enhance effective tumoricidal immunity but also induce long‐lasting immune memory effects. These results contribute to the inhibition of tumor progression, recurrence and lung metastasis, thereby prolonging the overall survival of tumor‐bearing mice. This work underscores the potential of an engineered biohybrid micromotor system as an alternative therapeutic approach in immunotherapy to enhance efficacy against tumors.

## Graphical abstract

Cryo‐inactivated cancer cell‐based magnetic micromotors are developed for synergistic tumor immunotherapy via magnetic targeting. These micromotors can induce ferroptosis and immunogenic cell death, deliver tumor antigens, and activate the Mn^2+^‐dependent cGAS‐STING pathway. The system effectively suppresses tumor progression, recurrence, and metastasis, prolongs survival, and establishes durable immune memory. These findings highlight an innovative biohybrid strategy to potentiate cancer immunotherapy.

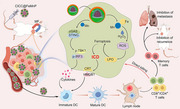

## 1 Introduction

Immunotherapy has gained recognition as a promising strategy for tumor control and eradication by activating and improving the immune system to discern and attack cancer cells.^[^ ^1^ ^]^ Currently, various immunotherapeutic approaches have achieved impressive progress, such as the blockade therapy of immune checkpoints, adaptive immune cell therapy, etc.^[^ ^2^ ^]^ Nevertheless, a limited proportion of cancer patients exhibit a response to contemporary immunotherapy, attributable to the low immunogenicity and the immunosuppressive nature of the tumor microenvironment.^[^ ^3^ ^]^ The presence of an immune‐deficiency feature in these tumors results in insufficient immune cells infiltrating into the tumor periphery or stroma.^[^ ^4^ ^]^ Besides, the immunosuppressive tumor microenvironment promotes tumor cells to evade the host immune response, thereby promoting tumor progression and metastasis.^[^ ^5^ ^]^ Therefore, developing new therapeutic platforms to tackle these challenges for enhancing anti‐tumor immunotherapy is still anticipated.

Micromotor carriers are devices capable of transforming electrical, chemical, or other forms of energy into mechanical energy to facilitate the movement of tiny objects or systems.^[^ ^6^ ^]^ Due to their diminutive size, efficient energy conversion, and versatile modes of operation, micromotor carriers hold significant potential for application across various domains, especially in the biomedicine field.^[^ ^7^ ^]^ Recently, cell‐based microcarriers have gained prominence as a promising research focus within the domains of biomedicine and drug delivery, attributed to their excellent biocompatibility, functional diversity, modifiability, structural integrity, and the availability of various types.^[^ ^8^ ^]^ These systems have been utilized to deliver therapeutic agents, encompassing engineered immune cells, stem cells, erythrocytes, platelets, and adipocytes, demonstrating significant potential for tumor therapy.^[^ ^9^ ^]^ For example, Ci et. al. developed therapeutic “dead cells” by freezing acute myeloid leukemia (AML) cells in liquid nitrogen (LNT cells), eliminating their pathogenicity while preserving structural integrity.^[^ ^8b^ ^]^ In AML mice, LNT cells improved doxorubicin delivery to bone marrow and functioned as a cancer vaccine, boosting antitumor immunity. Deng and colleagues engineered a biohybrid system (Bc@AZTF) to enhance immunogenic cell death (ICD) while blocking immunosuppressive adenosine production.^[^ ^9a^ ^]^ The Bc@AZTF consumes intracellular adenosine triphosphate (ATP) to inhibit adenosine formation and amplify ICD. When combined with an immune checkpoint inhibitor, Bc@AZTF enhanced antitumor immunity, established immune memory, and prevented tumor recurrence. However, the challenges related to inadequate target ability and limited retention within the tumor region substantially undermine the therapeutic efficacy of the loaded drugs. Therefore, it is conceived that by integrating micromotors with cell‐based microcarriers, multifunctional therapeutic systems could be developed to improve tumor immunotherapy efficiently.

**Figure 1:**
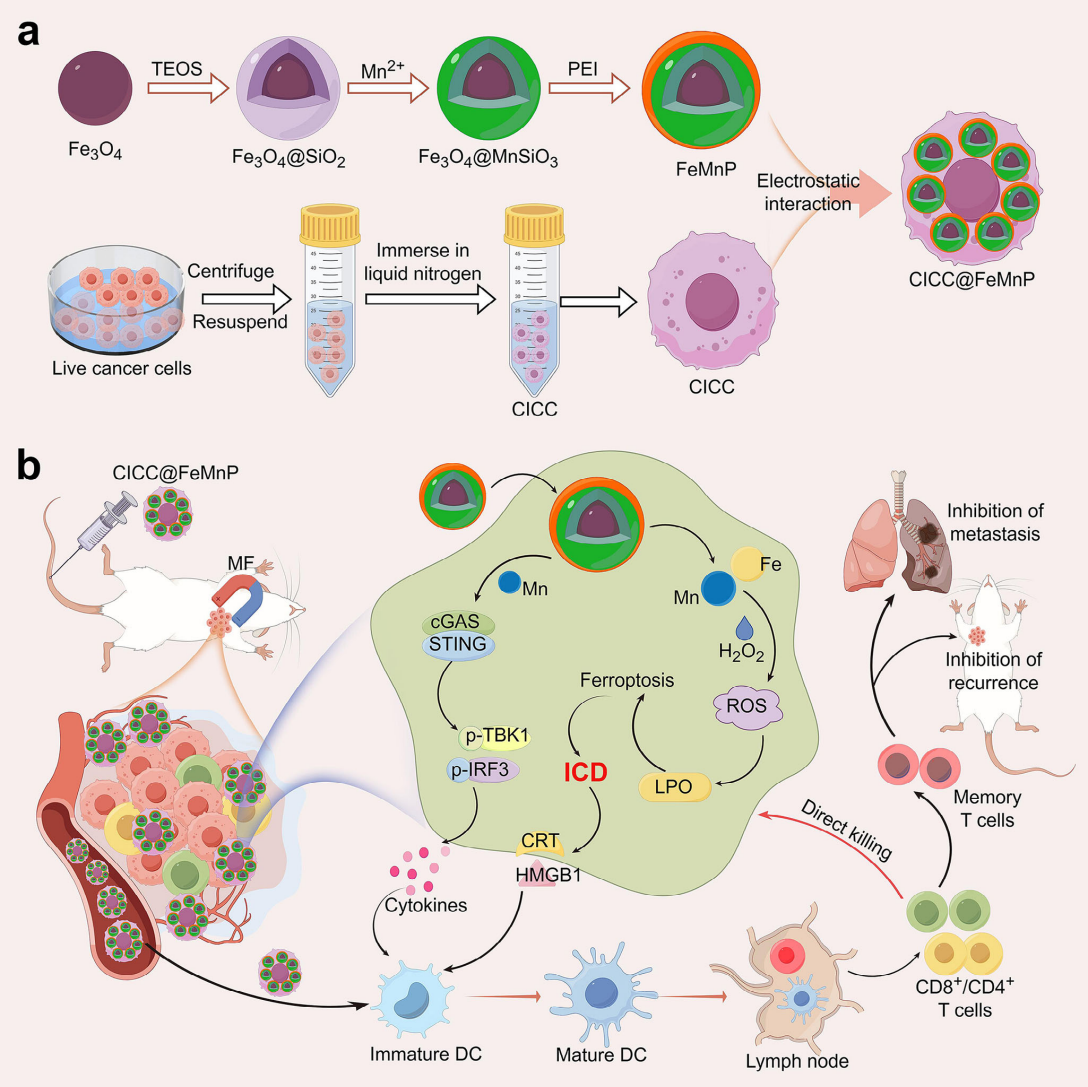
Schematic of engineered biohybrid micromotors (CICC@FeMnP) for tumor immunotherapy. a) Preparation processes of FeMnP and CICC and the fabrication of CICC@FeMnP. b) The therapeutic mechanism of CICC@FeMnP micromotor‐mediated anti‐tumor immunotherapy. The diagram was created by Figdraw.

To implement this conception, we present novel cryo‐inactivated cancer cells (CICC) derived magnetic micromotors (CICC@FeMnP) with the desired features for effective tumor immunotherapy, as schemed in **Figure** **1**. The cancer cells were inactivated by the cryo‐shocking method based on liquid nitrogen treatment. The CICC preserved their structural integrity, enabling the facile modification of magnetic nanoparticles FeMnP onto their surface to produce CICC@FeMnP micromotors. Remarkably, the engineered CICC@FeMnP micromotors exhibited magnetically controlled behavior that facilitates therapeutic component enrichment to target sites. Besides, the FeMnP could initiate Fenton reactions to induce ferroptosis, thereby triggering immunogenic cell death (ICD). In addition, the FeMnP could enhance the body's immune response through the Mn^2+^‐mediated cyclic GMP‐AMP synthase‐stimulator of interferon genes (cGAS‐STING) pathway. When integrated with the CICC, which carries a diversity of tumor antigens, an immunogenic microenvironment could be achieved. Thus, in the established orthotopic breast tumor model, the CICC@FeMnP micromotors could significantly suppress tumor progression, recurrence, and lung metastasis, benefiting mice's survival. These findings indicated that the engineered biohybrid magnetic micromotors represent an innovative and adaptable strategy in cancer immunotherapy, offering significant potential for advancements in the field of tumor therapy.

## 2 Results and Discussion

**Figure 2:**
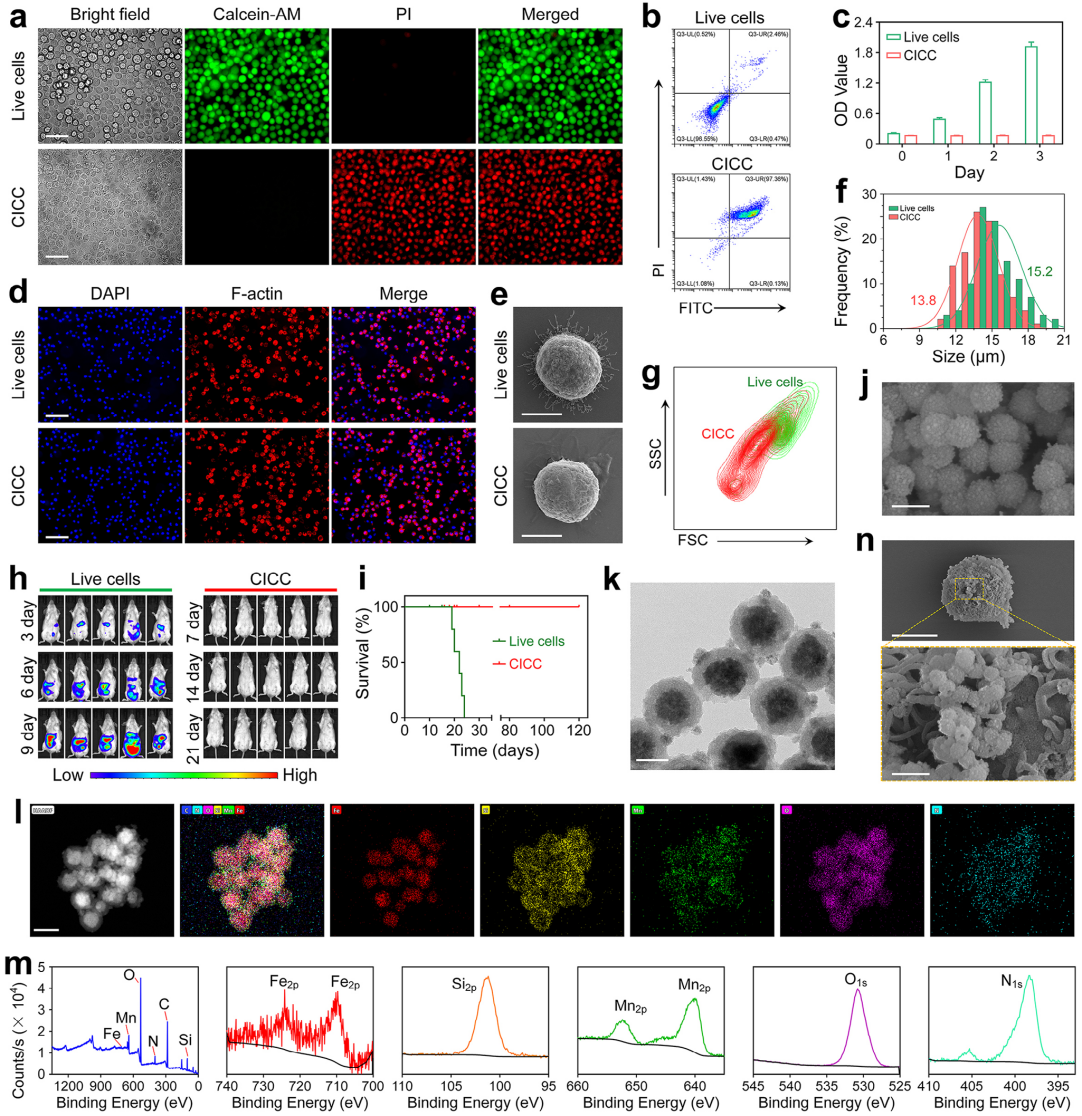
Characterization of CICC, FeMnP, and CICC@FeMnP. a) Live/dead staining images of live cells and CICC. Scale bars, 50 µm. b) FCM analysis of live cells and CICC. c) Cell viability of live cells and CICC using the CCK8 assay. d) Cell structure images of live cells and CICC. Scale bars, 50 µm. e) SEM images of live cells and CICC. Scale bars, 5 µm. f) Cellular sizes of live cells and CICC. g) FCM analysis of live cells and CICC under the same voltages. h) The bioluminescence signals of mice following the injection of live cells and CICC tagged with luciferase (*n* = 5). i) The survival of mice after being challenged with 1 × 10^6^ live cells and CICC (*n* = 5). j) SEM images of FeMnP. Scale bar, 250 nm. k) TEM images of FeMnP. Scale bar, 100 nm. l) Elemental mapping of FeMnP. Scale bar, 200 nm. m) XPS spectra of FeMnP. n) SEM images of CICC@FeMnP. Scale bars, 5 µm (up), 0.5 µm (down).

In a typical experiment, mouse breast cancer 4T1 cells were inactivated through a freeze‐thaw cycle, where they were submerged in liquid nitrogen for 10 min and then thawed at 37 °C to obtain the inactivated cancer cells. After the liquid nitrogen treatment, nearly all the cells were dead as observed by live/dead staining and flow cytometry (FCM) analysis (**Figure** **2**a,b; Figure S1, Supporting Information). Besides, the proliferative activity of cancer cells inactivated by liquid nitrogen was studied using the cell counting kit‐8 (CCK8) assay. Compared with the live cells, the CICC did not show proliferative activity as shown in Figure 2c. Under the microscopy observation, the CICC exhibited comparable cellular morphology to untreated live cells as evidenced by staining of the nucleus and cytoskeleton (Figure 2d). Besides, the sodium dodecyl sulfate–polyacrylamide gel electrophoresis (SDS‐PAGE) assay showed that the CICC preserved nearly all the typical proteins expressed in live 4T1 cells (Figure S2, Supporting Information). As observed by scanning electron microscopy (SEM), CICC exhibited a spherical morphology with a rough surface resembling that of live cells, suggesting that the structural integrity of cancer cells was maintained during the freeze‐thaw process (Figure 2e). A modest reduction in cellular dimensions was noted, as illustrated in Figure 2f, with CICC displaying an average size of 13.8 µm and untreated live cells measuring 15.2 µm. The forward scatter (FSC) values obtained through FCM supported the reduction in cell size of CICC (Figure 2g). To investigate the pathogenic potential of CICC, cryo‐inactivated and live 4T1 cells were administered to mice via intraperitoneal injection, with subsequent monitoring of oncogenic progression. As shown in Figure 2h,i, the results showed a notable rise of live 4T1 cells in vivo over 9 days, as indicated by the strong bioluminescence signal. Conversely, no bioluminescence signal was observed in mice injected with CICC even at day 21, and all mice survived for a minimum of 120 days. These findings suggested that CICC exhibits biosafety both in vitro and in vivo.

Subsequently, uniform magnetic Fe_3_O_4_@MnSiO_3_ nanoparticles were synthesized via a one‐step hydrothermal method. Briefly, Fe_3_O_4_ nanoparticles were first prepared according to previous reports and characterized by SEM, dynamic light scattering (DLS), and Fourier transform infrared spectrum (FT‐IR) (Figures S3–S5, Supporting Information). Subsequently, the magnetic nanoparticles underwent a coating process with silica dioxide (SiO_2_) using the modified Stöber method,^[^ ^10^ ^]^ resulting in the formation of a SiO_2_ shell layer (Figures S3–S5, Supporting Information). In an alkaline solution at high temperature, the SiO_2_ shell promotes the interaction between silicate ions and Mn ions, which results in the decoration of MnSiO_3_ on the surface of magnetic Fe_3_O_4_. As shown in Figures S3–S6 (Supporting Information), the Fe_3_O_4_@MnSiO_3_ exhibited an average diameter of ≈250 nm, consisting of magnetic cores measuring ≈150 nm in diameter, and spherical shells with a thickness of ≈50 nm. The FT‐IR spectra also indicated the presence of Fe_3_O_4_, specifically Fe_3_O_4_@SiO_2_ and Fe_3_O_4_@MnSiO_3_ nanoparticles (Figure S5, Supporting Information). The results demonstrated a shift in vibration peaks of Fe─O bonds from 550 to 480 cm^−1^ after SiO_2_ coating. Additionally, new vibrational peaks appeared at 840 and 996 cm^−1^, along with a pronounced peak at 1120 cm^−1^. These peaks are attributed to the functional groups Si─O─Si, Si─OH, and Si─O, respectively. Furthermore, most of the characteristic peaks associated with the silica layer disappeared upon the formation of Fe_3_O_4_@MnSiO_3_, transforming into a single vibration peak at 1070 cm^−1^. Moreover, the elemental compositions of various nanoparticles were confirmed through the analysis of X‐ray photoelectron spectroscopy (XPS) results (Figure S7, Supporting Information). The magneto‐responsive nature of Fe_3_O_4_@SiO_2_ and Fe_3_O_4_@MnSiO_3_ nanoparticles was investigated. As shown in Figure S8 (Supporting Information), the nanoparticles suspended in an aqueous solution could be concentrated using an external magnetic field (MF).

Next, Fe_3_O_4_@MnSiO_3_ with negative charge (−18.7 mV) was coated with polyethyleneimine (PEI) to obtain the positively charged nanoparticles (Fe_3_O_4_@MnSiO_3_@PEI, denoted as FeMnP, 28.8 mV) (Figure S9, Supporting Information). The SEM and transmission electron microscopy (TEM) images revealed that the PEI coating exerted minimal influence on the particle size and surface morphology of the nanoparticles (Figure 2j,k). The results of the elemental mapping analysis demonstrated that Si, Mn, O, and N were distributed uniformly on the shells of FeMnP, while Fe was predominantly localized within the core (Figure 2l). XPS results also indicated the elemental compositions of the FeMnP (Figure 2m). These results demonstrated the successful preparation of FeMnP. Then, the degradation and release behavior of FeMnP under different conditions were investigated. The TEM analysis revealed that FeMnP remained stable for up to 48 h at a pH of 7.4 (Figure S10, Supporting Information). In contrast, a gradual degradation occurred at pH 5.4. Notably, under the observation of TEM, the originally relatively dense MnSiO_3_ shell became more transparent after gradually degrading. It was observed that at a pH of 7.4, the release of Mn and Fe from FeMnP was slow, with only 10.99% and 7.22% being released after 48 h, respectively (Figure S11, Supporting Information). In contrast, under acidic conditions at pH 5.4, the release contents of Mn and Fe significantly increased to 55.91% and 39.79%, respectively. These findings suggest that FeMnP exhibits acid sensitivity, maintaining its structure under normal physiological conditions while releasing metal ions in the acidic tumor microenvironment, facilitating further therapeutic effects.

Subsequently, cell‐nanoparticle biohybrids (i.e., CICC@FeMnP) were fabricated by integrating the positively charged FeMnP with the negatively charged CICC, facilitated by electrostatic interactions. As shown in the SEM images, FeMnP was effectively decorated onto the surface of CICC (Figure 2n). The magnetic hysteresis loops of Fe_3_O_4_, FeMnP, and CICC@FeMnP demonstrated their superparamagnetic behavior. Despite a noticeable reduction in the saturation magnetization value of CICC@FeMnP, attributed to the complexation of FeMnP with CICC, the material retained its magnetic responsiveness (Figure S12, Supporting Information). The X‐ray diffraction (XRD) analysis of FeMnP demonstrated that the crystallographic structure of the core magnetic Fe_3_O_4_ was preserved following the decoration with MnSiO_3_ shells and complexation with PEI (Figure S13, Supporting Information). The CICC@FeMnP exhibited XRD peaks analogous to those of Fe_3_O_4_ and FeMnP, suggesting that the complexation of FeMnP with CICC did not alter their composite or crystal structure.

**Figure 3:**
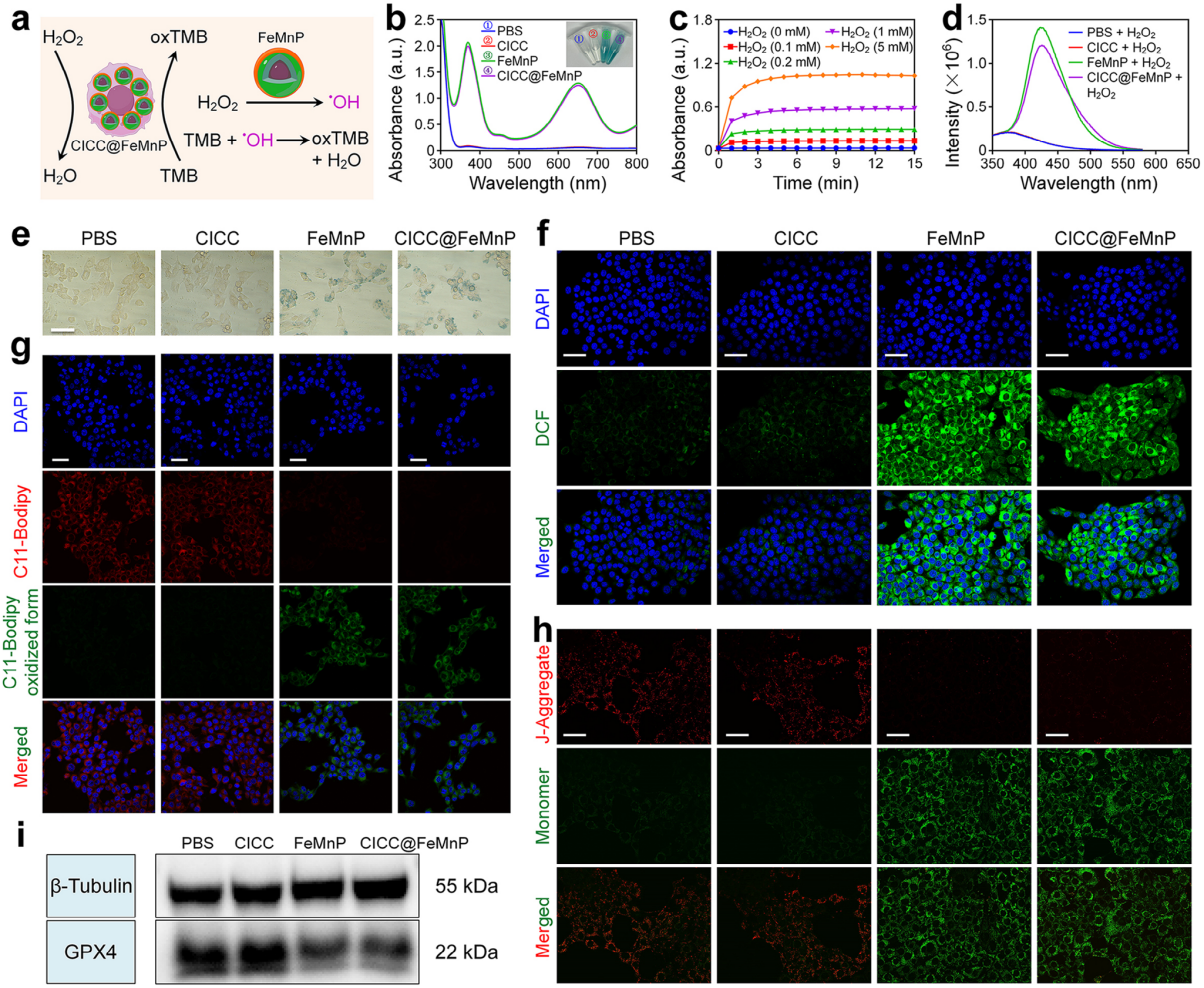
Ferroptosis‐induce ability of CICC@FeMnP. a) Schematic presentation of ^•^OH induced by CICC@FeMnP and the oxidation processes of TMB. b) UV–vis absorption spectra of the oxidized TMB (oxTMB) under various conditions. The insets illustrate the corresponding visual color changes. c) The absorbance changes of TMB after treatment with CICC@FeMnP and H_2_O_2_ with different concentrations. d) Fluorescence spectra of PPTA with different treatments. e) Prussian blue staining of 4T1 cells after various treatments for 8 h. Scale bar, 25 µm. f) CLSM images of 4T1 cells after different treatments and incubated with DCFH‐DA. Scale bars, 50 µm. g) CLSM images of 4T1 cells after different treatments and incubated with C11‐BODIPY^581/591^. Scale bars, 50 µm. h) Fluorescence images of mitochondrial membrane potential detections of 4T1 cells after different treatments, as revealed by the JC‐1 probe. Scale bars, 50 µm. i) WB analysis of GPX4 protein in 4T1 cells after incubation with various formulations.

As is well‐known, Fe_3_O_4_ is a classical nano‐enzyme, and Fe^2+^ and Mn^2+^ have Fenton‐like activities.^[^ ^11^ ^]^ Therefore, the FeMnP could catalyze H_2_O_2_ decomposition to generate hydroxyl radical (^•^OH), as shown in **Figure** **3**a. To verify this, tetramethylbenzidine (TMB) and *p*‐Phthalic acid (PPTA) were applied to detect the ^•^OH generation. As illustrated in Figure 3b, through the chromogenic reaction and absorbance spectra of TMB, FeMnP and CICC@FeMnP incubated with H_2_O_2_ could produce ^•^OH to oxidize TMB. The elevated absorption value of TMB at 652 nm was positively correlated with the augmented concentration of H_2_O_2_ (Figure 3c). PPTA could react with ^•^OH to form a strong fluorescent substance 2‐hydroxyterephthalic acid, which is highly selective to ^•^OH.^[^ ^12^ ^]^ Thus, in the presence of PPTA, FeMnP and CICC@FeMnP reacting with H_2_O_2_ could result in a significant enhancement of the fluorescence intensity of PPTA (Figure 3d; Figure S14, Supporting Information), implying the substantial production of ^•^OH in the reaction solution.

Following these studies, we evaluated the cellular uptake of FeMnP and CICC@FeMnP by 4T1 cells via Prussian blue staining analysis. As observed in Figure 3e, the blue dye was clearly seen in the cells incubated with FeMnP and CICC@FeMnP, in contrast to the control group and those treated with CICC. This suggests the successful delivery of the nanoagents. Subsequently, the intracellular reactive oxygen species (ROS) generation induced by CICC@FeMnP was investigated using the 2’,7’‐dichlorodihydrofluorescein diacetate (DCFH‐DA) indicator. As shown in Figure 3f, the green fluorescence from DCF in the FeMnP and CICC@FeMnP treatment groups could be observed in 4T1 cells. This should be ascribed to the nano‐enzyme activity and Fenton‐like reaction ability of FeMnP. In addition, C11‐BODIPY^581/591^ served as a marker to assess intracellular lipid peroxide (LPO) levels, a recognized indicator of ferroptosis,^[^ ^13^ ^]^ following induction by CICC@FeMnP. Confocal laser scanning microscopy (CLSM) images revealed varying degrees of LPO in 4T1 cells treated with different agents (Figure 3g). Notably, cells in the FeMnP and CICC@FeMnP treatment groups demonstrated enhanced green fluorescence from the oxidized C11‐BODIPY^581/591^, attributable to ROS generation, thereby indicating the significant ferroptosis‐inducing capacity of CICC@FeMnP. Mitochondria are vulnerable to damage under high intracellular ROS levels.^[^ ^14^ ^]^ Therefore, mitochondrial damage was assessed by measuring alterations in the membrane potential of mitochondria using the JC‐1 probe. CLSM images revealed that 4T1 cells subjected to PBS treatment, as well as those treated with CICC, exhibited red fluorescence indicative of JC‐1 aggregates, signifying an intact mitochondrial membrane potential (Figure 3h). In contrast, minimal red fluorescence was detected following treatment with FeMnP and CICC@FeMnP, while green fluorescence reached its maximum intensity. This observation confirmed that the mitochondria of 4T1 tumor cells were significantly compromised following these treatments. Glutathione peroxidase 4 (GPX4) protein was considered to be an important ferroptosis marker because it could effectively eliminate cellular ROS and then avoid ferroptosis.^[^ ^14^, ^15^ ^]^ Therefore, the GPX4 expression levels were analyzed. As illustrated in Figure 3i, there was a significant reduction in GPX4 expression following CICC@FeMnP treatment, thereby confirming the occurrence of ferroptosis.

**Figure 4:**
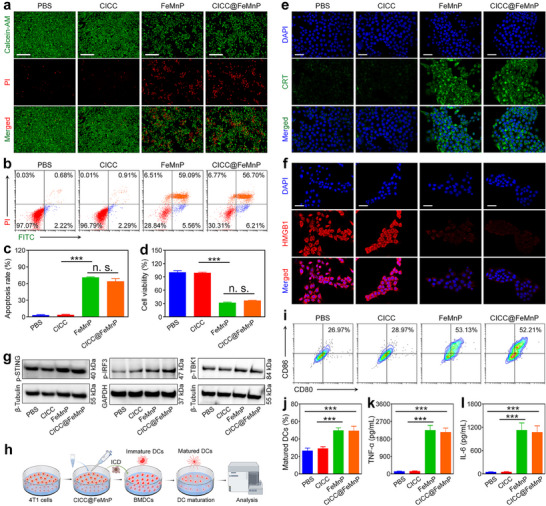
In vitro anti‐cancer efficacy of CICC@FeMnP and DC maturation. a) Live/dead staining images of 4T1 cells after different treatments. Scale bars, 100 µm. (b, c) FCM analysis b) and corresponding apoptosis rates c) of 4T1 cells after various treatments. d) Analysis of 4T1 cell viability following various treatments using the CCK8 assay. e,f) CRT (e) and HMGB1 (f) expression of 4T1 cells after various treatments by immunofluorescence staining. Scale bars, 50 µm. g) WB analysis of p‐STING, p‐IRF3, and p‐TBK1 expression in 4T1 cells incubated with various formulations. h) Schematic diagram of ICD and DC maturation processes. i,j) FCM analysis of DC maturation (i) and corresponding maturation ratios (j) after various treatments. k,l) Quantitative assessment of cytokine secretion (TNF‐α and IL‐6) by DCs. Data are presented as the mean ± SD. n. s.: no significance, ^***^ *p* < 0.001.

Building upon the aforementioned mechanisms, the therapeutic efficacy of CICC@FeMnP was evaluated in vitro against 4T1 cells. A live/dead cell staining assay was employed to assess the cytotoxicity of CICC@FeMnP. As observed in **Figure** **4**a, CICC displayed negligible influence on the proliferation of 4T1 cells, while FeMnP and CICC@FeMnP exhibited appreciable killing effects against cancer cells. Additionally, the results of FCM showed the same trend. Specifically, the apoptosis rates of 4T1 cells were measured as follows: 3.10 ± 1.08% in the control group, 3.50 ± 1.07% in the CICC group, 70.95 ± 1.13% in the FeMnP group, and 63.88 ± 5.05% in the CICC@FeMnP group (Figure 4b,c). Moreover, the CCK8 assay indicated a significant cytotoxic effect of FeMnP and CICC@FeMnP on cancer cells (Figure 4d). These results demonstrate that FeMnP effectively induces tumor cell death, and this tumor cell‐killing capability is maintained when FeMnP is incorporated into the CICC@FeMnP composite system.

Based on prior research demonstrating that ferroptosis can effectively induce ICD across various tumor cell types,^[^ ^16^ ^]^ the capacity of CICC@FeMnP to induce ICD in 4T1 cells was meticulously assessed. This evaluation involved analyzing the release profiles of damage‐associated molecular patterns (DAMPs), including calreticulin (CRT), high mobility group box 1 (HMGB1), and ATP. As expected, the treatment of FeMnP and CICC@FeMnP significantly promoted the presentation of CRT on the cancer cell membranes and facilitated the extracellular release of HMGB1 (Figure 4e,f). Besides, the levels of extracellular ATP were significantly increased following treatment of the cells with FeMnP and CICC@FeMnP (Figure S15, Supporting Information). These results indicated the potent induction of ICD by the complex system. Given the substantial presence of Mn^2+^ ions in CICC@FeMnP, we assessed the potential of CICC@FeMnP to initiate the signaling pathway of cGAS‐STING. Western blot (WB) analysis indicated significant increases in the phosphorylation levels of STING, interferon regulatory factor‐3 (IRF3), and TANK‐binding kinase 1 (TBK1) proteins (denoted as p‐STING, p‐IRF3, and p‐TBK1) following treatment with FeMnP and CICC@FeMnP (Figure 4g). The elevated contents of p‐IRF3 and p‐TBK1 suggest the activation of the STING pathway. These results indicate that CICC@FeMnP functions as an efficient Mn^2+^ delivery system, capable of triggering the STING pathway in tumor cells to enhance innate immune responses. The DAMPs and STING activation may facilitate the maturation of dendritic cells (DCs). To validate this, supernatants from various treatment conditions were collected and added to immature DCs (Figure 4h). As anticipated, the proportion of mature DCs in the FeMnP and CICC@FeMnP treatment groups demonstrated a statistically significant increase relative to the control and CICC groups (Figures 4i,j). Moreover, the secretion levels of tumor necrosis factor‐alpha (TNF‐α) and interleukin‐6 (IL‐6) in the CICC@FeMnP treatment cells were markedly elevated, exhibiting elevations of 17.98‐fold and 24.34‐fold, respectively, compared to the control group (Figure 4k,l). Collectively, these results demonstrate that the synthesized CICC@FeMnP has the potential to synergistically induce robust ICD and activate the STING pathway, thereby promoting DC maturation. The dual mechanism, wherein ferroptosis‐driven ICD provides antigenic signals and Mn^2+^‐mediated STING activation enhances innate immune recognition, establishes a self‐reinforcing loop that augments antitumor immunity. This facilitates both the eradication of tumor cells and the maintenance of long‐term immune surveillance. The integration of these pathways in the CICC@FeMnP system highlights its ability to exploit redox dysregulation and metal ion‐mediated immunomodulation to achieve robust immune activation.

**Figure 5:**
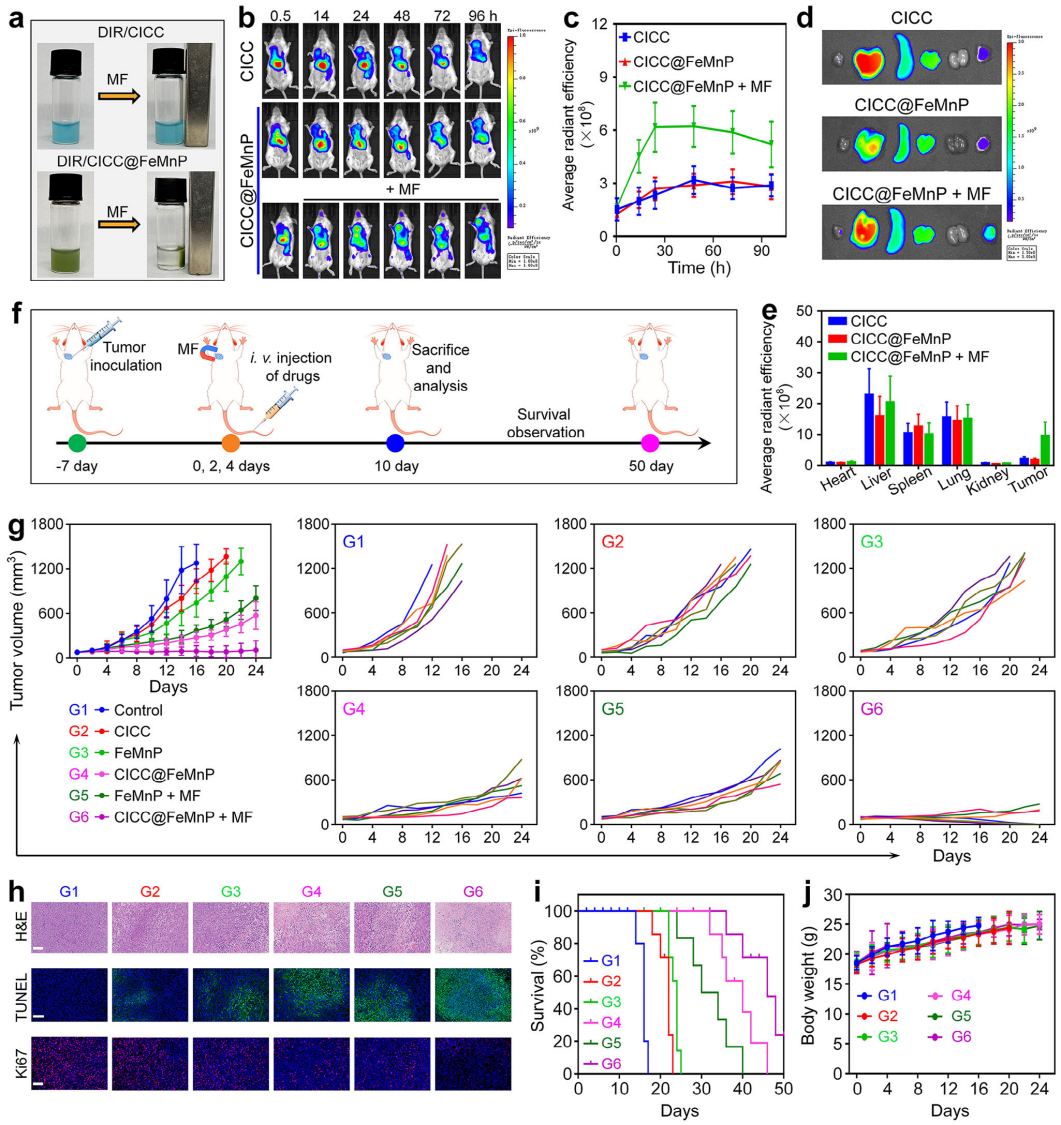
Magnetic control behavior and in vivo anti‐cancer efficacy of CICC@FeMnP. a) Images of DIR‐labeled CICC or CICC@FeMnP with or without MF guidance. b) Representative in vivo fluorescence images of tumor‐bearing mice after intravenous injection of DIR‐labeled CICC and CICC@FeMnP, with or without MF. c) The signal intensity within the tumor regions from in vivo images. d) Ex vivo fluorescence images of organs and tumors after different treatments for 96 h. e) The signal intensity within the organs and tumor regions from ex vivo images. f) Treatment schedule of Balb/c mice bearing 4T1 orthotopic tumors treated by CICC@FeMnP combined with MF. g) Tumor growth curves of mice treated with different formulations (*n* = 6). h) H&E, TUNEL, and Ki67 staining of isolated tumor tissues after the treatments. Scale bars, 100 µm. i) Survival rate analysis of mice in different groups (*n* = 6). j) Body weight changes of mice in different groups during the treatment processes (*n* = 6).

Attributed to the enhanced magnetic properties of FeMnP decorated on the CICC, the CICC@FeMnP could also exhibit magnetism, allowing their movements to be directed under magnetic influence. Significantly, this magnetic polarity ensured that the CICC@FeMnP consistently aligned with the direction of the MF. Both DIR‐labeled and unlabeled CICC@FeMnP were maneuvered using a strong magnet to illustrate their magnetic responsiveness. As shown in **Figures** **5**a and S16 (Supporting Information), CICC@FeMnP could effectively move and concentrate on the wall of a vial near the magnet. Besides, the Movies S1 and S2 (Supporting Information) provided a directed magnetic response motion of CICC@FeMnP. The DIL‐labeled CICC or CICC@FeMnP was placed in a well, and after 15 s, an MF was applied to the right side of the well. As observed in Movie S2 (Supporting Information), the CICC@FeMnP exhibited directional movement in response to the applied MF. In contrast, the CICC remains stationary regardless of whether an MF was applied (Movie S1, Supporting Information). The ability to effectively target tumors is crucial for achieving optimal anti‐tumor effects.^[^ ^17^ ^]^ To evaluate in vivo precise magnetic targeting efficiency of CICC@FeMnP, DIR‐labeled CICC and CICC@FeMnP were employed to track their biodistribution in mice with 4T1 tumors. The labeling efficiency of DIR was measured to be 93.5% according to the UV–vis spectroscopy (Figure S17, Supporting Information). This high efficiency contributed to the tracking of the micromotor system in the biodistribution studies. Following intravenous administration, a gradual increase in fluorescence was observed at the tumor area from 0.5 to 24 h post‐injection for both DIR‐labeled CICC and CICC@FeMnP, without an applied magnetic field (MF). Notably, in the group of CICC@FeMnP plus MF, the fluorescence intensity continued to increase more significantly 24 h post‐injection (Figure 5b,c; Figure S18, Supporting Information). Besides, the fluorescence signal at tumor areas persisted at a relatively high level following the injection of CICC@FeMnP combined with MF after 24 h, compared to the other two groups, demonstrating the superior magnetic targeting capability and effective retention of CICC@FeMnP at tumor sites. Furthermore, major organs and tumors were surgically removed 96 h after injection for ex vivo imaging (Figure 5d,e). Notably, the average radiant efficiency at tumor areas in mice injected with CICC@FeMnP under MF was distinctly higher than that in the CICC and CICC@FeMnP groups without MF, further corroborating the enhanced magnetic control ability and enrichment of CICC@FeMnP in tumor tissues.

Given its promising therapeutic efficacy demonstrated in vitro and its effective magnetic targeting capability observed in vivo, an orthotopic breast tumor mouse model was employed to examine the anti‐tumor efficacy of CICC@FeMnP. According to the treatment protocol outlined in Figure 5f, mice were inoculated with 4T1 tumors and subsequently administered intravenous injections of Saline, CICC, FeMnP, and CICC@FeMnP on days 0, 2, and 4, with or without the application of MF. The tumor size and body weight of the mice that received different treatments were systematically measured. Analysis of the tumor volumes revealed that, relative to the control group, all other treatment modalities displayed differing levels of tumor growth inhibition (Figure 5g). Notably, the treatment involving CICC@FeMnP in conjunction with MF markedly suppressed the growth of tumors, resulting in the smallest tumor volume observed among all treatment groups. Concurrently, the efficacy of each treatment in eradicating tumors was assessed by analyzing tumor sections using hematoxylin and eosin (H&E) staining, along with Ki67 and terminal‐deoxynucleotidyl transferase‐mediated nick end labeling (TUNEL) immunofluorescence assays. The results from these analyses confirmed that the CICC@FeMnP combined with MF treatment overtly inhibited growth and proliferation, induced apoptosis and necrosis, and destroyed the structural integrity of tumor cells (Figure 5h). This combined treatment not only reduced the tumors but also extended the mice's survival (Figure 5i). Throughout the intervention process, the body weights of the mice subjected to various treatments showed a significant increase (Figure 5j). Additionally, the biosafety of the prepared formulations was also confirmed through histological examination of major organs using H&E staining. The results indicated no pathological changes in these organs after different treatments, thereby demonstrating the in vivo biosafety of the CICC@FeMnP formulations (Figure S19, Supporting Information).

**Figure 6:**
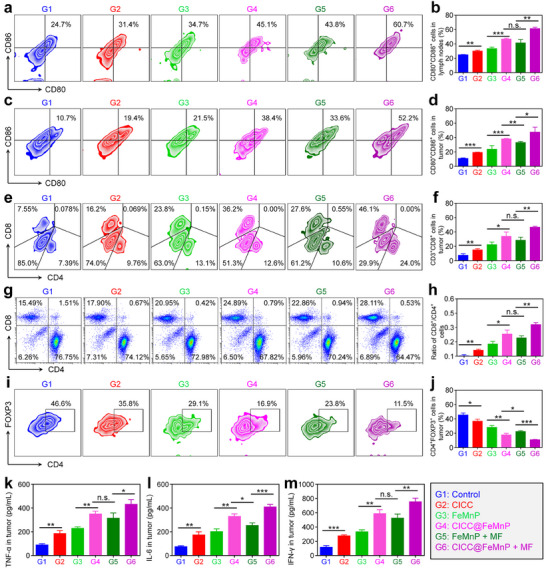
Immune evaluation. a,b) FCM analysis (a) and corresponding quantitative data (b) of CD80^+^CD86^+^ cells among CD11c^+^ cells in LNs. c,d) FCM analysis (c) and corresponding quantitative data (d) of CD80^+^CD86^+^ cells among CD11c^+^ cells in tumors. e,f) FCM analysis (e) and corresponding quantitative data (f) of CD8^+^ T cells among CD3^+^CD45^+^ T cells in tumors. g,h) FCM analysis (g) and corresponding quantitative data (h) of CD8^+^/CD4^+^ T cells ratio in the spleen. i,j) FCM analysis (i) and corresponding quantitative data (j) of CD4^+^Foxp3^+^ cells among CD3^+^CD45^+^ T cells in tumors. k–m) Cytokine levels of TNF‐α (k), IL‐6 (l), and IFN‐γ (m) in tumors. Data are presented as the mean ± SD. n. s.: no significance, ^*^ *p* < 0.05, ^**^ *p* < 0.01, ^***^ *p* < 0.001, ^****^ *p* < 0.0001.

Then, the anti‐tumor mechanisms of CICC@FeMnP were investigated. The roles and proportions of various immune cells within the tumor microenvironment (TME) significantly influence tumor development. In many solid tumors, exemplified by the 4T1 breast tumor, the TME is frequently featured by immunosuppressive properties and exhibits resistance to immunotherapeutic interventions. Consequently, the response and infiltration of diverse immune cells within tumor tissues were examined to elucidate the tumor inhibition mechanisms of CICC@FeMnP from an immunotherapeutic perspective. Initially, the capacity of CICC@FeMnP to induce ICD was assessed in 4T1 tumors on day 10 by measuring the levels of classical ICD markers, HMGB1 and CRT (Figure S20, Supporting Information). As anticipated, immunofluorescence staining from the tumor sections demonstrated that FeMnP‐related treatment could obviously enhance the expression of CRT and release of HMGB1. Notably, the CICC@FeMnP + MF treatment elicited the most pronounced effects among the formulations tested, effectively inducing ICD in tumor cells. The potential of CICC@FeMnP‐mediated ICD to trigger anti‐tumor immunity was assessed by evaluating the DC maturation in the draining lymph nodes (LNs) and tumor tissues using FCM. This is crucial as mature DCs are integral in presenting antigens to T lymphocytes, thereby evoking anti‐tumor immune responses. According to the FCM results, treatments with CICC@FeMnP and CICC@FeMnP + MF significantly increased the percentages of mature DCs in both LNs and tumor tissues compared to the control group, suggesting efficient antigen capture and presentation (**Figure** **6**a–d).

Given that DC maturation represents the initial phase of anti‐tumor immunity, it is hypothesized that CICC@FeMnP could exhibit significant potential in immunotherapeutic applications. To evaluate this hypothesis, the proportions of CD8^+^ T cells within tumor tissues were investigated to determine the capacity of CICC@FeMnP to enhance immunotherapy. Consistent with expectations, the CICC@FeMnP + MF intervention led to a marked enhancement in the ratio of CD8^+^ T lymphocytes at tumor sites, 6.19 times greater than those recorded in the control group (Figure 6e,f). This finding underscores the efficacy of CICC@FeMnP in conjunction with MF in augmenting immunotherapeutic outcomes. A parallel trend was observed in the spleens, where a significantly elevated CD8^+^/CD4^+^ ratio was noted following CICC@FeMnP + MF‐mediated immunotherapy (Figure 6g,h). Meanwhile, the proportions of immunosuppressive regulatory T cells (Tregs) within tumors were assessed, revealing a significant decrease in CD3^+^CD4^+^Foxp3^+^ Tregs to 10.87% following treatment with CICC@FeMnP + MF (Figure 6i,j). This represented a 4.18‐fold decrease compared to the control group, which exhibited a Treg population of 45.47%. Moreover, the content of cytokines (TNF‐α, IL‐6, and IFN‐γ) in tumors was assessed following various treatments using enzyme‐linked immunosorbent assay (ELISA) (Figure 6k–m). Both the standalone CICC@FeMnP treatment and the combined CICC@FeMnP + MF treatment significantly elevated the intratumoral levels of cytokines, which are associated with immune activation.

**Figure 7:**
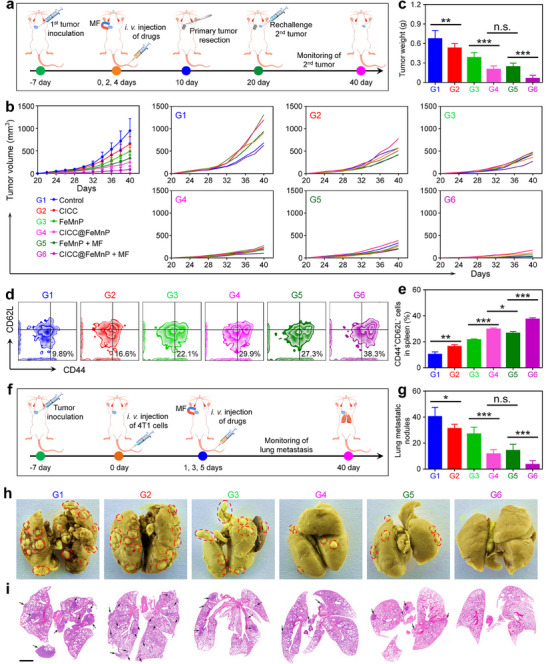
Anti‐tumor effects on preventing 4T1 tumor recurrence and lung metastasis. a) Diagrammatic representation of treatment cycles for assessing the anti‐recurrent efficacy. b) Tumor growth curves of mice subjected to different treatment formulations (*n* = 6). c) Weights of recurrent tumors excised following diverse treatment regimens (*n* = 6). d,e) FCM analysis (d) and corresponding quantitative data (e) of CD44^+^CD62L^−^ T_em_ cells among CD3^+^CD8^+^ T cells in spleens. f) Diagrammatic representation of treatment cycles for assessing the anti‐metastatic efficacy. g) Lung metastatic nodules of each group. h) Representative lung images of each group, with red circles indicating metastatic sites. i) Representative H&E‐stained lung sections of each group, with black arrows highlighting metastatic regions. Scale bar, 2 mm. Data are presented as the mean ± SD. n. s.: no significance, ^*^ *p* < 0.05, ^**^ *p* < 0.01, ^***^ *p* < 0.001, ^****^ *p* < 0.0001.

The recurrence of solid tumors after drug intervention remains a significant challenge, underscoring the critical need for effective strategies to mitigate tumor recurrence within oncology.^[^ ^18^ ^]^ Previous results have shown that CICC@FeMnP combined with MF‐based multimodal therapy can elicit robust anti‐tumor immune responses. Consequently, we explored the potential of CICC@FeMnP to induce a durable anti‐tumor immune memory ability, which could effectively inhibit the proliferation of rechallenged homologous cancer cells in murine models. The treatment processes are schemed in **Figure** **7**a. The tumor‐bearing mice received various treatments. Following a 10‐day treatment period, we performed surgical removal of the initial tumor that had previously achieved remission through various treatments. Then, a rechallenged second tumor was inoculated at day 20. As depicted in Figure 7b,c, and Figure S21 (Supporting Information), the CICC@FeMnP + MF treatment made both the size and weight of the rechallenged tumors a marked decrease. These results indicate that the CICC@FeMnP + MF‐based treatment strategy is capable of generating a synergistic action, which may induce long‐term immune memory and provide protection against tumor recurrence in murine models. To understand the mechanisms behind anti‐tumor immune memory, spleens were collected from mice after a 19‐day treatment. FCM analysis was then used to measure the CD8^+^ effector memory T (*T*_em_) cell populations. As illustrated in Figure 7d,e, the CICC@FeMnP + MF treatment strategy could potentially increase the population of *T*_em_ cells (CD3^+^CD8^+^CD44^+^CD62L^−^) in the spleen. By further activating these cells, they can differentiate into cytotoxic T lymphocytes, thus effectively stopping tumor recurrence.

Given the well‐documented propensity of the 4T1 breast tumor for metastasis, a 4T1 lung metastasis model was developed to study the efficacy of CICC@FeMnP combined with MF treatment in inhibiting metastatic progression. To establish lung metastasis foci, 4T1 cells were administered intravenously via the tail vein and subsequently subjected to the treatment regimen outlined in the accompanying Figure 7f. Lungs from mice were harvested on day 40 following tumor cell inoculation, and the metastatic lesions were quantified. As depicted in Figure 7g, the treatment with FeMnP, CICC@FeMnP, FeMnP + MF, and CICC@FeMnP + MF decreased the number of lung metastatic nodules. Specifically, the control group exhibited a mean of 40.3 ± 7.1 metastatic nodules per lung. In contrast, treatment with FeMnP, CICC@FeMnP, and FeMnP + MF reduced the mean metastatic nodule number to 27.0 ± 5.2, 11.7 ± 3.3, and 14.3 ± 4.8 per lung, respectively. Notably, the CICC@FeMnP + MF treatment resulted in an average of only 3.5 ± 3.0 metastatic nodules per lung. The inhibition of metastatic nodules was clearly demonstrated through photographic evidence and H&E staining of lung tissue (Figure 7h,i). The alveolar spaces in the CICC@FeMnP + MF treatment were evidently clearer in comparison to the control group, and the metastatic nodules were fewer and smaller than those observed in other treatments. These findings indicate that the CICC@FeMnP + MF treatment exhibited the most pronounced anti‐metastatic effects relative to the other groups.

## 3 Conclusion

In summary, we developed a magnetic micromotor system (CICC@FeMnP) as an effective therapeutic platform for synergistic tumor immunotherapy. The process of liquid nitrogen treatment effectively eliminates the tumorigenic potential of tumor cells while maintaining the cellular architecture integrity. This preservation of CICC facilitates the subsequent loading of CICC with magnetic nanoparticles, thereby endowing the CICC@FeMnP with a controllable capacity for tumor targeting. The FeMnP‐mediated ferroptosis could induce ICD of tumor cells. Together with various tumor antigens from CICC, an immunogenic tumor microenvironment could be achieved. In addition, Mn^2+^ from FeMnP could promote the body's immune response through the cGAS‐STING pathway. Thus, the CICC@FeMnP demonstrates a significant enhancement in anti‐tumor, anti‐recurrence, and anti‐metastasis efficacy in vivo. This is substantiated by observations of delayed tumor progression, inhibition of recurrence, a decrease in metastatic foci in the lungs, and extended overall survival in murine models. These results indicated that the engineered biohybrid micromotor is a promising therapeutic system for tumor immunotherapy. Although the micromotor systems demonstrate promising biomedical functionalities, their translational development remains incomplete without a systematic evaluation of biological persistence, particularly concerning long‐term toxicity, bioaccumulation, and metabolic profiling. These aspects need to be investigated in future research endeavors.

## 4 Experimental Section

### Materials

D‐Luciferin potassium salt (D‐LPS), manganese chloride monohydrate (MnCl_2_•H_2_O), 3,3’,5,5’‐tetramethylbenzidine dihydrochloride hydrate (TMB•HCl), *p*‐Phthalic acid (PPTA) were bought from Macklin. DCFH‐DA reactive oxygen species assay kit, CCK8 assay kit, mitochondrial membrane potential assay kit with JC‐1, annexin V‐FITC/PI apoptosis detection kit were purchased from Beyotime Biotechnology Co., Ltd. Prussian blue stain kit was obtained from Solarbio Science & Technology Co., Ltd. C11‐BODIPY^581/591^ was bought from Invitrogen. Anti‐APC/Cy7‐CD45 antibody, anti‐FITC‐CD11c antibody, anti‐PE‐CD80 antibody, and anti‐APC‐CD86 antibody, anti‐FITC‐CD3, anti‐APC‐CD4, anti‐PE‐CD8a, anti‐PE‐Foxp3, anti‐APC‐CD44, and anti‐AF/700‐CD62L^−^ were bought from Biolegend. GAPDH polyclonal antibody and β‐Tubulin polyclonal antibody were bought from Boster. Phosphor‐STING (ser366) polyclonal antibody, phosphor‐IRF3 (ser396) polyclonal antibody, and phosphor‐TBK1/NAK (ser172) polyclonal antibody were bought from Biodragon.

### Preparation and Characterization of CICC

1 × 10^7 of 4T1 cells were collected and diluted in PBS, and then cryopreserved in liquid nitrogen for 10 min. Prior to utilization, the cryopreserved cells were thawed in an ice bath, resuspended at 37 °C, subjected to centrifugation, and subsequently washed two times using PBS. To analyze the cellular structure, CICC were stained using DAPI and AF555‐conjugated phalloidin. Specifically, 1 × 10^6 CICC were incubated in PBS containing phalloidin stock solution for 20 min, followed by centrifugation. The cells were then stained with DAPI, followed by resuspended in PBS, and observed using CLSM. For structural observation, CICC was fixed in 2.5% glutaraldehyde for 30 min. Thereafter, dehydration was performed using a graded ethanol series with concentrations of 50%, 60%, 70%, 80%, and 90%, with each step lasting 10 min. This was followed by two washes in 100% ethanol, each for a duration of 30 min. The dehydrated CICC was subsequently placed on a silicon wafer for further analysis using SEM.

### Cell Proliferation of CICC

For the in vitro analysis of cell viability, 4T1 cells subjected to liquid nitrogen were stained with calcein‐AM and PI for 30 min, followed by examination using a CLSM (Evident/FV3000). Additionally, cell viability post cryo‐shock with liquid nitrogen was assessed using the CCK8 assay and FCM.

For in vivo cell proliferation studies, 2×10^6 either viable or liquid nitrogen‐treated luciferase‐tagged 4T1 cells were administered into the abdominal cavity of mice. The proliferation of viable cells was tracked by measuring bioluminescence signals on days 3, 6, and 9, while the proliferation of CICC was monitored on days 7, 14, and 21. Following an intraperitoneal injection of the substrate D‐LPS (150 mg kg^−1^), bioluminescence imaging was conducted via a multi‐mode optical in vivo imaging system (PerkinElmer) after 10 min.

### Protein Expression of CICC

The expression of whole‐cell proteins was assessed through SDS‐PAGE. Proteins were isolated from both viable and liquid nitrogen‐treated 4T1 cells using the RIPA lysis and protein extraction kit (Beyotime), with the addition of a protease inhibitor cocktail (Beyotime). After the determination of protein concentrations, samples were prepared with a sample buffer to ensure a protein load of 20 µg per well. Before SDS‐PAGE analysis, the proteins in the samples were denatured by heating to 95 °C for 10 min. The gel post‐electrophoresis was then stained with Coomassie blue and photographed with a digital camera.

### Preparation of CICC@FeMnP

The Fe_3_O_4_ and Fe_3_O_4_@SiO_2_ were first synthesized according to the previous reports.^[^ ^19^ ^]^ Then, a solution of 0.1672 g of NH_4_Cl and 36 mg of MnCl_2_•H_2_O in 10 mL of deionized water was prepared, followed by adding 0.39 mL of NH_3_•H_2_O. After mixing with 0.1 g of Fe_3_O_4_@SiO_2_ and stirring for 30 min, the mixture was then transferred to an autoclave and received thermal treatment at 140 °C for 14 h. Afterward, the system was naturally cooled to the environment temperature. The Fe_3_O_4_@MnSiO_3_ product was subsequently isolated using an external MF and washed thrice with deionized water. The Fe_3_O_4_@MnSiO_3_ was incubated with a polyethyleneimine (PEI, 0.5 mg mL^−1^) solution for 2 h to obtain PEI‐coated magnetic nanoparticles Fe_3_O_4_@MnSiO_3_@PEI, denoted as FeMnP. Subsequently, 1 × 10^7 CICC were combined with FeMnP (1 mg mL^−1^) and gently shaken for 1 h. The mixture was then collected using an external MF and washed with PBS to obtain CICC@FeMnP.

### Measurement of ^•^OH

TMB and PPTA were used to detect the ^•^OH generation. For TMB measurement, a mixture containing CICC (1 × 10^6), FeMnP (0.1 mg mL^−1^), or CICC@FeMnP (1 × 10^6) was introduced into a solution comprising 1mL of acetic acid (0.1 m, pH 5.0), TMB at a concentration of 1 mm, and varying concentrations of H_2_O_2_. Following co‐incubation for varying time intervals, absorbance at 652 nm was subsequently detected utilizing both a microplate reader and a UV–vis spectrometer. For PPTA measurement, the CICC, FeMnP, or CICC@FeMnP was combined with a PPTA solution (5 mm), which also contained NaOH (5 mm) and H_2_O_2_ (10 mm). After a 30 min incubation period, fluorescence spectra (325 to 600 nm) were obtained utilizing an excitation wavelength of 300 nm.

### Prussian Blue Staining Measurement

4T1 cells (2.5 × 10^5 cells per well) cultured in the 6‐well plate were exposed to CICC (1 × 10^6), FeMnP (0.1 mg mL^−1^), or CICC@FeMnP (1 × 10^6) for 8 h. After incubation, the treated cells were rinsed to eliminate non‐endocytosed materials. Thereafter, a Prussian blue solution was administered to stain the different treated cells for 30 min. Following this, the cells underwent three washes with PBS to eliminate any excess dye, fixed with paraformaldehyde, and visualized using a fluorescence microscope (ZEISS, Axio Vert.A1).

### Intracellular ROS and Lipid Peroxide Detection

4T1 cells (5 × 10^4 cells per well) cultured in the 24‐well plate were added CICC (1 × 10^6), FeMnP (0.1 mg mL^−1^), or CICC@FeMnP (1 × 10^6) and co‐incubated for 8 h. To detect ROS, a freshly prepared solution of DCFH‐DA at a concentration of 10 µm was administered to the treated cells for an incubation period of 30 min. Then the obtained cells were imaged by a CLSM. For the detection of lipid peroxides, cells subjected to various treatments were stained with C11‐BODIPY581/591 (10 µm) for 30 min, and were analyzed using the CLSM system.

### In Vitro GPX4 Analysis by WB

4T1 cells were incubated in the 6‐well plate. Following various treatments, 100 µL of RIPA Lysis Buffer supplemented with PMSF and PSIM at a ratio of 100:1:2 was added to the cells. The cells were subsequently collected into 1.5 mL centrifuge tubes and incubated on ice for 10 min. Next, the cells were subjected to centrifugation at 12 000 rpm for 15 min at 4 °C, after which the supernatant was collected for protein concentration determination using a BCA assay kit. Prior to assessing the expression levels of specific proteins via WB analysis, the samples were heated to 95 °C for 10 min. The primary antibodies employed included a β‐Tubulin polyclonal antibody (Boster) and GPX4 (Boster), which were incubated for 12 h at 4 °C. This was followed by three washes with 10 mm TBST and subsequent incubation with secondary antibodies. Finally, protein bands were visualized using a Bio‐Rad Chemidox XRS gel imaging system in conjunction with an ECL substrate.

### Mitochondrial Membrane Potential Measurement

4T1 cells were subjected to the aforementioned treatments and subsequently incubated with JC‐1 for 20 min via a mitochondrial membrane potential assay kit. After the incubation, the cells were examined using a CLSM.

### In Vitro Anti‐Tumor Effects

4T1 cells (4000 cells per well) incubated in a 96‐well plate were treated with CICC (1 × 10^6), FeMnP (0.1 mg mL^−1^), or CICC@FeMnP (1 × 10^6). After a 24‐h incubation period, the CCK8 assay was performed. Cells treated for 24 h were cultured with Calcein‐AM and PI, then examined under a fluorescence microscope for live/dead analysis. In the apoptosis analysis by flow cytometry (FCM), cells were subjected to the same treatments as previously described, followed by administration with an apoptosis detection kit.

### In Vitro ICD Effect

4T1 cells (2.5 × 10^5 cells per well) were initially seeded and cultured overnight in a 6‐well plate. Subsequently, the cells were subjected to various treatments. Following an additional 24‐h incubation period, the cells underwent a series of washing, fixation, and blocking procedures for 20 min. Afterward, the cells were mixed with an antibody (anti‐BF488‐CRT, Bioss) for 30 min post‐washing. In contrast to the aforementioned staining method with anti‐BF488‐CRT, the procedure for staining with the anti‐BF555‐HMGB1 antibody (Bioss) included an additional step, namely 0.3% Triton X‐100. All images were acquired using CLSM. In addition, the extracellular level of ATP following various treatments was assessed using the enhanced ATP detection kit (Beyotime Biotechnology) to analyze ATP secretion by the cells.

### WB Assay

4T1 cells cultured in a six‐well plate underwent various treatments. Following incubation, 100 µL of RIPA Lysis Buffer with PMSF and PSIM at a 100:1:2 ratio was added. Subsequently, the cells were collected into the centrifuge tubes (1.5 mL) and incubated on ice for 10 min. Thereafter, the cells underwent centrifugation (12 000 rpm, 15 min, 4 °C), and the supernatant was collected for protein concentration measurement with a BCA assay kit. Before evaluating the expression levels of specific proteins by WB analysis, the samples were maintained at 95 °C for 10 min. The antibodies utilized included GAPDH polyclonal antibody (Boster), β‐Tubulin polyclonal antibody (Boster), phosphor‐STING (ser366) polyclonal antibody, phosphor‐IRF3 (ser396) polyclonal antibody, and phosphor‐TBK1/NAK (ser172) polyclonal antibody were incubated for 12 h at 4 °C, followed by three washes with 10 mm TBST and subsequent treatment with the secondary antibodies. Finally, protein bands were visualized with a Bio‐Rad Chemidox XRS gel imaging system and ECL substrate.

### In Vitro DC Maturation

To study DC maturation, BMDCs were isolated from BALB/c mice's tibiae and femurs, incubated for seven days, and then collected for further analysis. Meanwhile, 4T1 cells were treated with different treatments for 24 h as described above. Afterward, the supernatants from the treated tumor cells were introduced to the BMDCs and cultured for 24 h. The mature DCs were co‐cultured with antibodies: anti‐CD11c, anti‐CD80, and anti‐CD86. These cells were subsequently analyzed using FCM. The cytokines secreted by the mature DCs were quantified using ELISA kits provided by Boster.

### Magnetic Responsiveness of CICC@FeMnP

To observe the motion of CICC@FeMnP, the CICC was first stained with DIR or DIL and then complexed with FeMnP. Afterward, the DIR/CICC@FeMnP dispersed in PBS was attracted by MF and could be directly visualized in the vial. On the other hand, the MF‐mediated DIL/CICC@FeMnP motion was observed using a fluorescence microscope.

### In Vivo Imaging and Biodistribution

An orthotopic breast tumor model was obtained by injecting 1 million 4T1 cells into mice, producing tumors around 100 mm^3^. To examine the biodistribution of CICC and CICC@FeMnP, they were labeled with DiR. Subsequently, the mice were intravenously administered 100 µL of CICC (5 × 10^6) or CICC@FeMnP (5 × 10^6), with or without the application of an MF. Then, the mice were observed under an in vivo imaging instrument at various time intervals (Excitation: 740 nm, Emission: 790 nm). At 96 h post‐injection, the isolated organs and tumors were imaged. The animal experiments received approval from the Animal Ethics Committee of the Wenzhou Institute, University of Chinese Academy of Sciences (approval number: WIUCAS24122404).

### In Vivo Anti‐Tumor Effects

Mice with 4T1 tumors were allocated into six distinct groups, each comprising six mice, and administered intravenous injection treatments consisting of 100 µL of saline, CICC (1 × 10^6), FeMnP (0.1 mg mL^−1^), CICC@FeMnP (1 × 10^6), FeMnP (0.1 mg mL^−1^, with MF), and CICC@FeMnP (1 × 10^6, with MF) on days 0, 2, and 4. Mice's weights and tumors were detected every 2 days. After 16 days, mice were euthanized, and tumors and primary organs were preserved in 4% paraformaldehyde. These tissues were subsequently sliced into 5 µm sections for histological analyses, including H&E, TUNEL, and Ki67 staining.

### In Vivo Anti‐Tumor Immunity Evaluation

Mice with 4T1 tumors were administered various treatments and subsequently euthanized on day 10. Tumors, spleens, and lymph nodes were harvested, mechanically dissociated, and filtered to generate single‐cell suspensions. To conduct in vivo analysis of DC maturation, cells isolated from lymph nodes and tumors were initially treated with anti‐mouse CD16/32. After that, live/dead cell discrimination was performed with the Zombie Violet Fixable Viability Kit (Biolegend), followed by a 30‐min incubation at 4 °C with antibodies: anti‐CD45, anti‐CD11c, anti‐CD80, and anti‐CD86. Following staining, the cells were subjected to FCM to assess DC maturation. For the evaluation of T‐cell populations, single‐cell suspensions derived from tumors and spleens were initially blocked with anti‐mouse CD16/32 antibodies. This was followed by live/dead cell staining to differentiate viable cells from non‐viable ones. Cells were incubated for 30 min with anti‐CD45, anti‐CD3, anti‐CD4, and anti‐CD8a antibodies, then analyzed via FCM to evaluate CD4^+^ and CD8^+^ T cell populations. Tregs were analyzed by treating tumor cell suspensions with 0.1% Triton X‐100 for 10 min and co‐staining with fluorescent antibodies against CD45, CD3, CD4, and Foxp3. The cells were subsequently measured using FCM. In addition, ELISA measured cytokine IL‐6, IFN‐γ, and TNF‐α levels in tumor samples. The induction of ICD in vivo was further validated in mice bearing 4T1 tumors. After a 10‐day treatment regimen, tumors were removed, preserved, sectioned, and stained using immunohistochemistry with anti‐BF488‐CRT or anti‐BF555‐HMGB1 antibodies. Fluorescence microscopy was employed to capture the resulting fluorescence images.

### Anti‐Recurrence Evaluation

In the anti‐recurrence tumor model, when the initial tumors grew to ≈100 mm^3^, the mice received the specified treatments. On day 10, surgical excision of the initial tumors was performed on mice from each group. On day 20, spleens from three mice per group were collected to evaluate the population of T_EM_ cells. Lymphocytes were stained using anti‐APC/Cy7‐CD45, anti‐FITC‐CD3, anti‐PE‐CD8a, anti‐APC‐CD44 (Biolegend), and anti‐AF/700‐CD62L^−^ (Biolegend) antibodies. The proportion of T_EM_ cells (CD3^+^CD8^+^CD44^+^CD62L^−^) was measured using FCM. On day 20, mice were injected with 5 × 10^5 4T1 cells in the left breast for tumor rechallenge, and the secondary tumor volumes and weights were evaluated.

### Inhibition of Tumor Lung Metastasis

Mice bearing the 4T1 tumor were randomly allocated into six distinct groups. After seven days, a 4T1 lung metastasis model was established through the intravenous administration of 1 × 10^5 cells. Subsequently, the mice received three intravenous treatments with 100 µL of either saline, CICC (1 × 10^6), FeMnP (0.1 mg mL^−1^), CICC@FeMnP (1 × 10^6), FeMnP (0.1 mg mL^−1^, with MF), or CICC@FeMnP (1 × 10^6, with MF) on days 1, 3, and 5. The mice were euthanized on day 40 to quantify metastatic foci, and their lungs were collected and fixed in Bouin's solution. After taking representative photographs, the lungs were subjected to H&E staining.

### Statistical Analysis

All statistical data are expressed as the mean ± standard deviation (SD). Statistical evaluation was analyzed using unpaired Student's t‐test or one‐way ANOVA, and a p‐value < 0.05 was considered statistically significant. n. s.: no significance, ^*^ *p* < 0.05, ^**^ *p* < 0.01, ^****^ *p*< 0.0001, ^***^ *p*< 0.001.

## Conflict of Interest

The authors declare no conflict of interest.

## Author Contributions

Y.J.Z. conceived the conceptualization and designed the experiment. Q.F.Z. carried out the experiments and analyzed the data. Q.F.Z., G.Z.K., W.Z.L. and Y.J.Z. wrote the paper.

## Supporting information



Supporting Information

Supplemental Movie 1

Supplemental Movie 2

## Data Availability

The data that support the findings of this study are available from the corresponding author upon reasonable request.
