# Supplementary material for: Cryo‐Inactivated Cancer Cells Derived Magnetic Micromotors for Tumor Immunotherapy
Source: Adv Sci (Weinh). 2025 May 23;12(30):e04986. doi: 10.1002/advs.202504986 (PMC12376701; doi:10.1002/advs.202504986)
Supplement: Supplementary file 1 — Supporting Information [file ADVS-12-e04986-s001.docx]

Supporting Information

**Cryo-Inactivated Cancer Cells Derived Magnetic Micromotors for Tumor Immunotherapy**

*Qingfei Zhang, Gaizhen Kuang, Wenzhao Li, Yuanjin Zhao**

Dr. Q. Zhang, Dr. G. Kuang, Dr. W. Li, Prof. Y. Zhao

Department of General Surgery, The First Affiliated Hospital of Wenzhou Medical University, Wenzhou 325035, China

E-mail: yjzhao@seu.edu.cn

Dr. Q. Zhang, Dr. G. Kuang, Dr. W. Li, Prof. Y. Zhao

Wenzhou Institute, University of Chinese Academy of Sciences, Wenzhou 325001, China

Prof. Y. Zhao

Department of Rheumatology and Immunology, Nanjing Drum Tower Hospital, School of Biological Science and Medical Engineering, Southeast University, Nanjing 210096, China


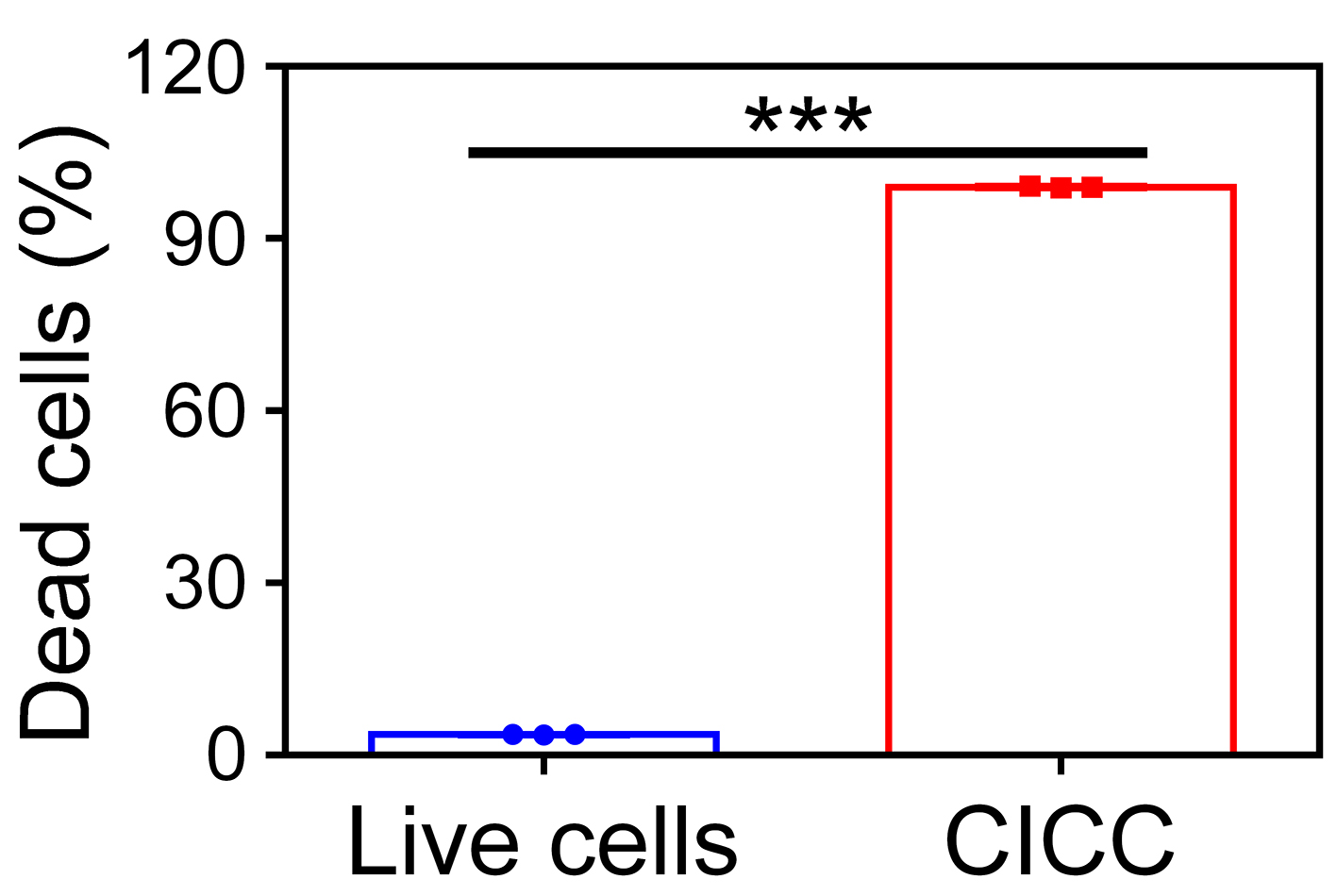


**Figure S1.** Quantitative data on dead cells before and after liquid nitrogen treatment.


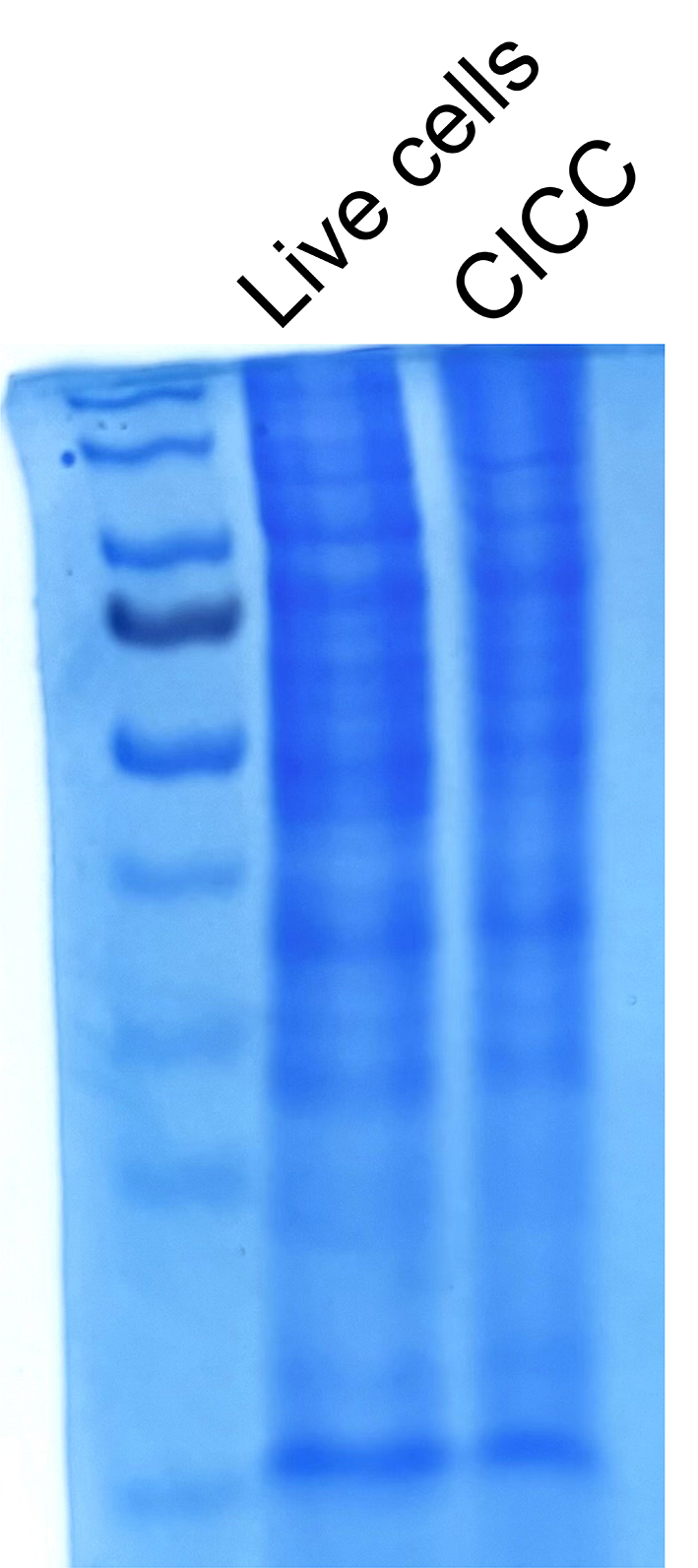


**Figure S2.** SDS-PEGA protein electrophoresis analyses of Live cells and CICC.


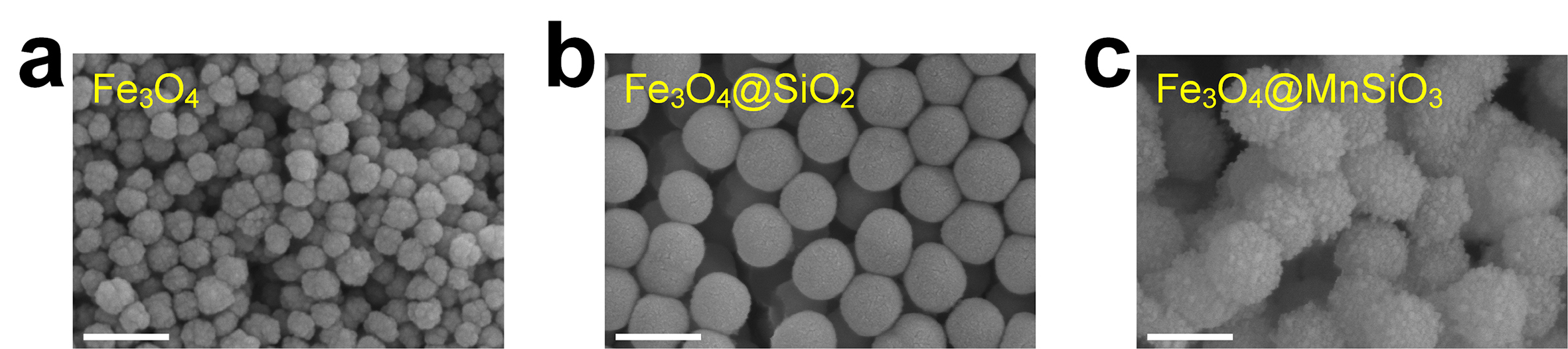


**Figure S3.** SEM images of Fe_3_O_4_ (a), Fe_3_O_4_@SiO_2_ (b), and Fe_3_O_4_@MnSiO_3_ (c). Scale bars, 250 nm.


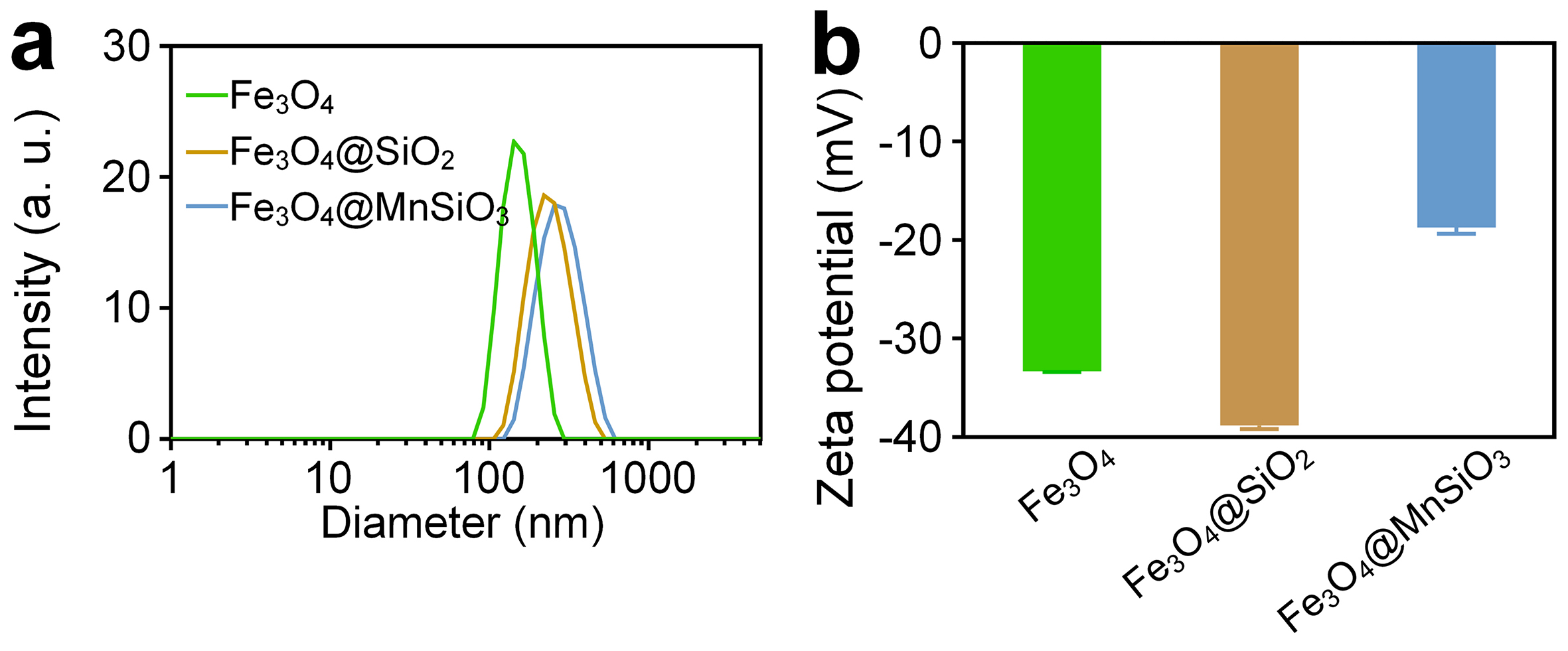


**Figure S4.** (a) Size distributions and (b) zeta potentials of Fe_3_O_4_, Fe_3_O_4_@SiO_2_, and Fe_3_O_4_@MnSiO_3_.


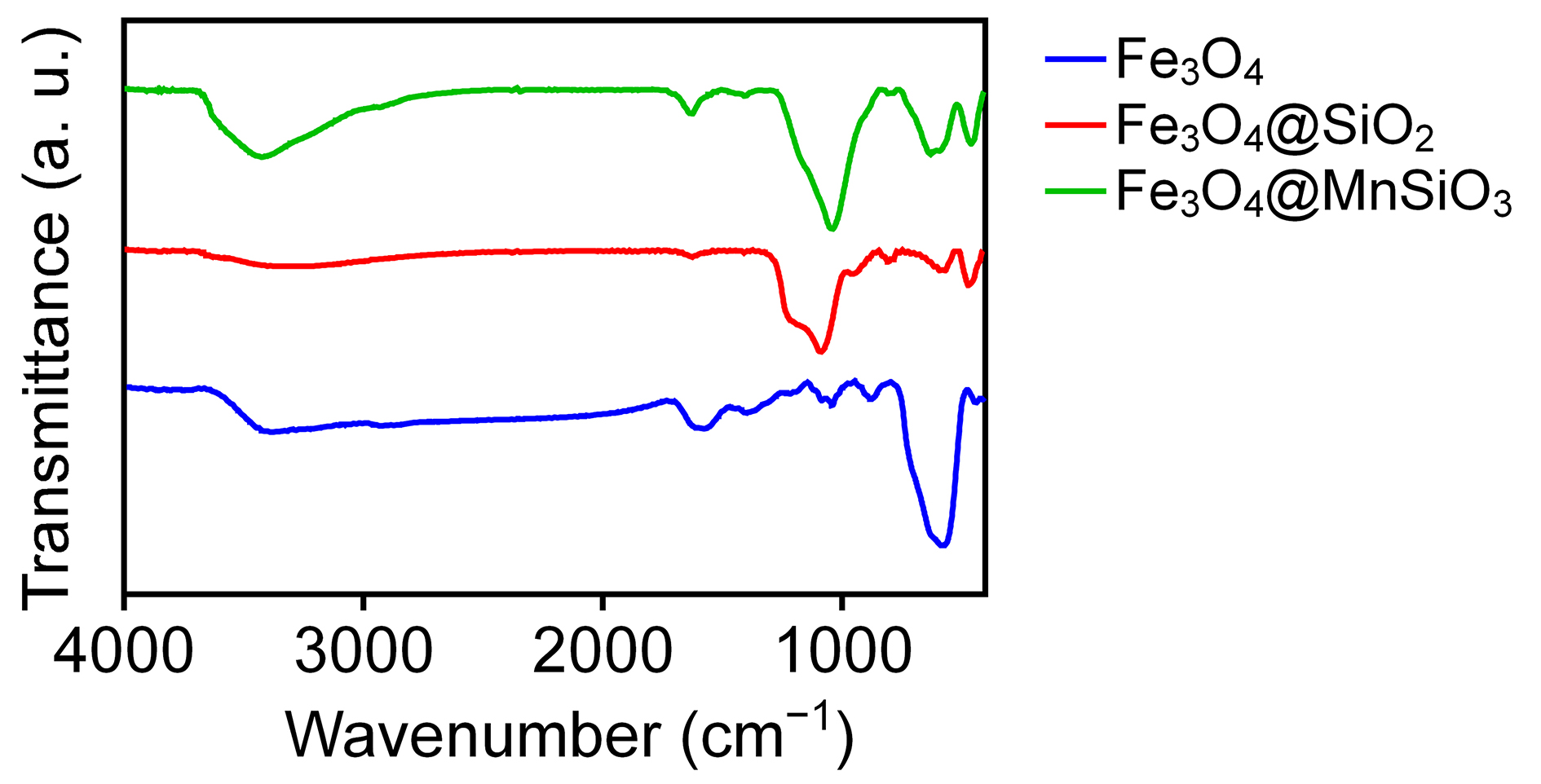


**Figure S5.** FT-IR of Fe_3_O_4_, Fe_3_O_4_@SiO_2_, and Fe_3_O_4_@MnSiO_3_.


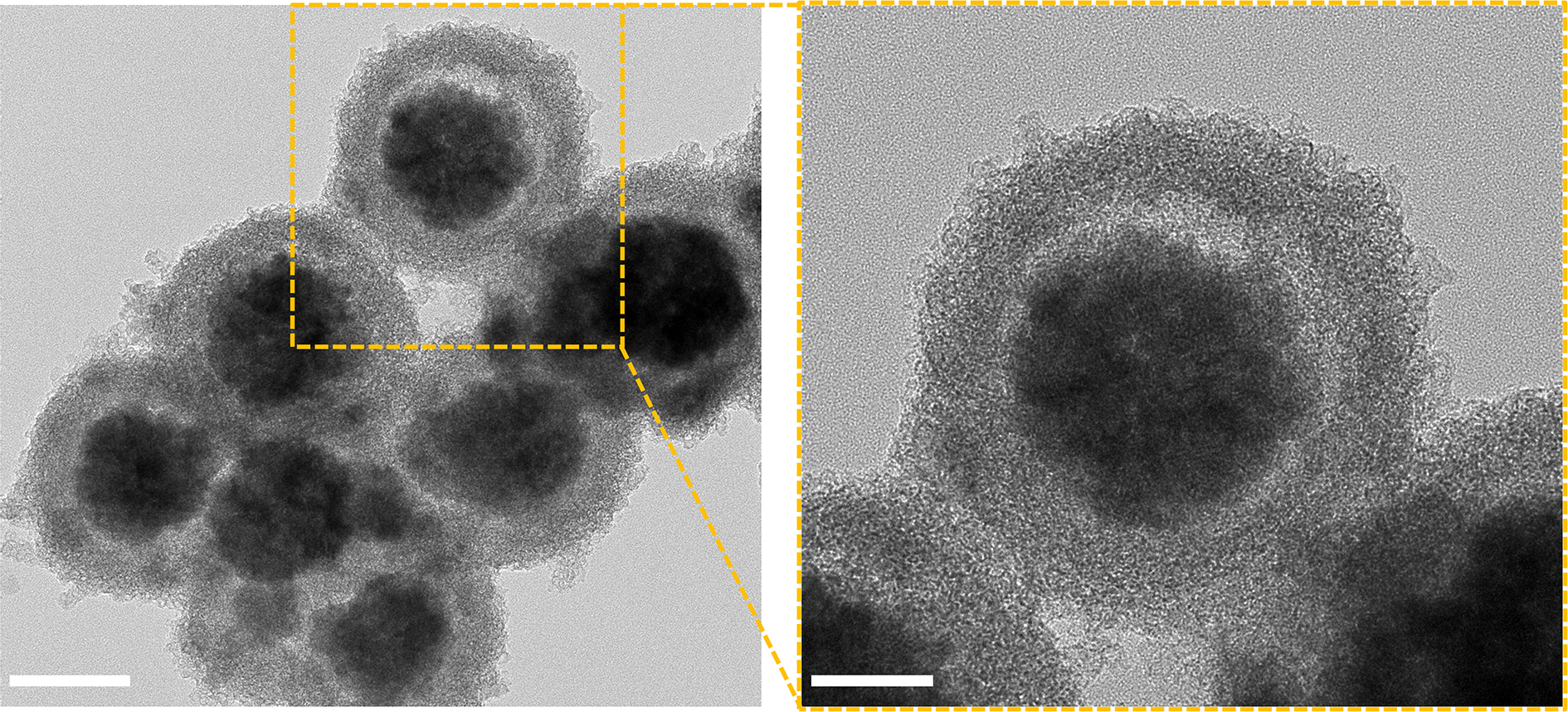


**Figure S6.** TEM images of Fe_3_O_4_@MnSiO_3_. Scale bars, 100 nm (left), 50 nm (right).


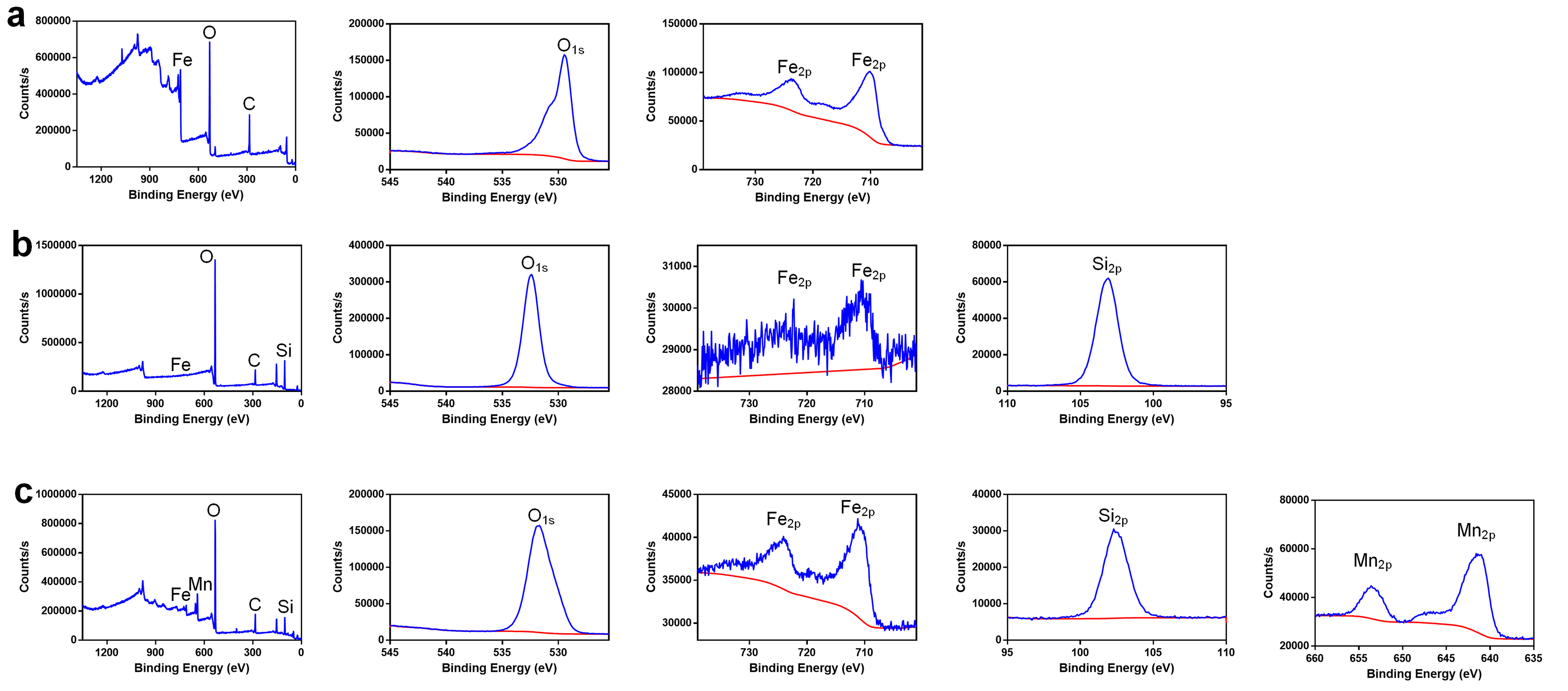


**Figure S7.** EDS spectra of Fe_3_O_4_ (a), Fe_3_O_4_@SiO_2_ (b), and Fe_3_O_4_@MnSiO_3_ (c).


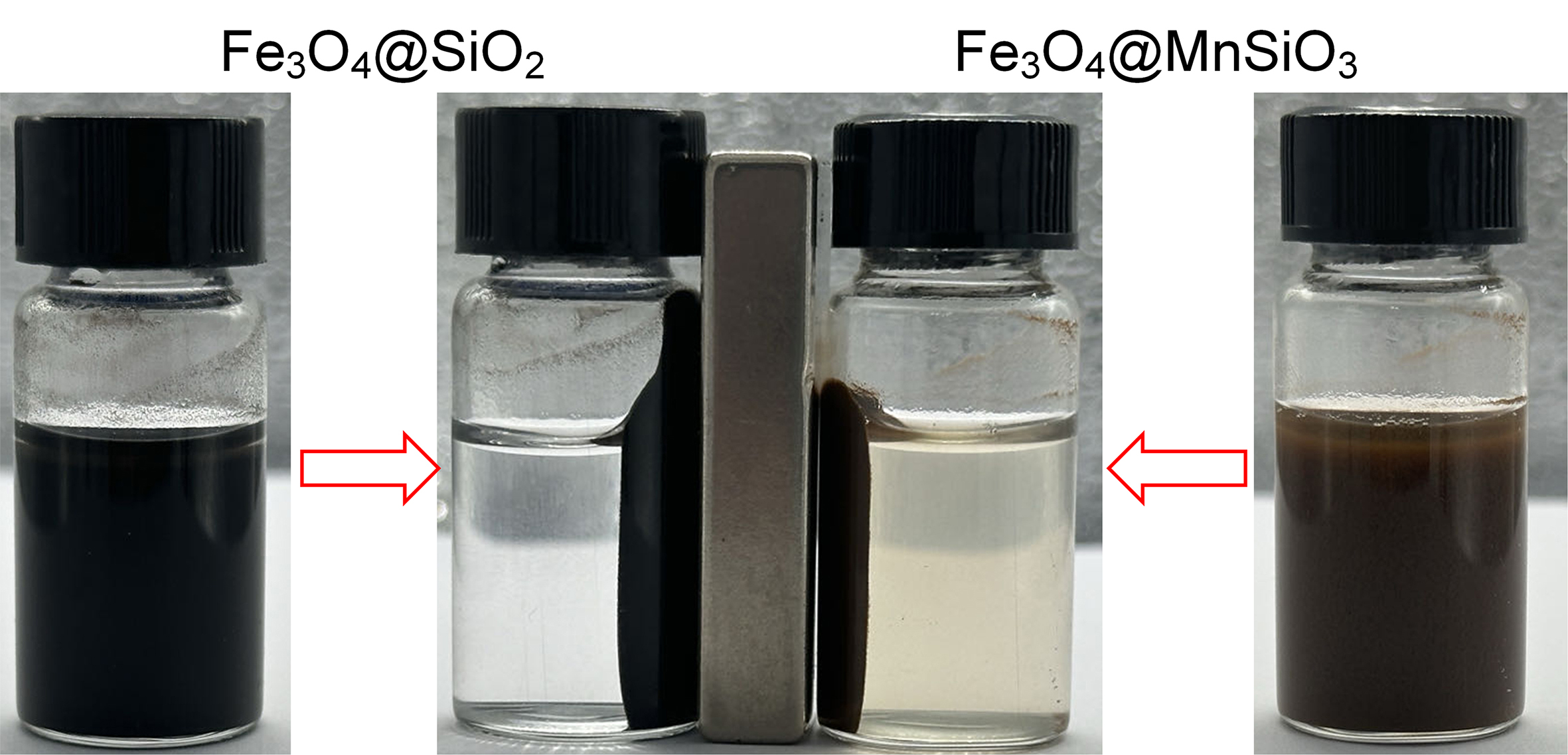


**Figure S8.** Images of Fe_3_O_4_@SiO_2_ and Fe_3_O_4_@MnSiO_3_ before and after MF action.


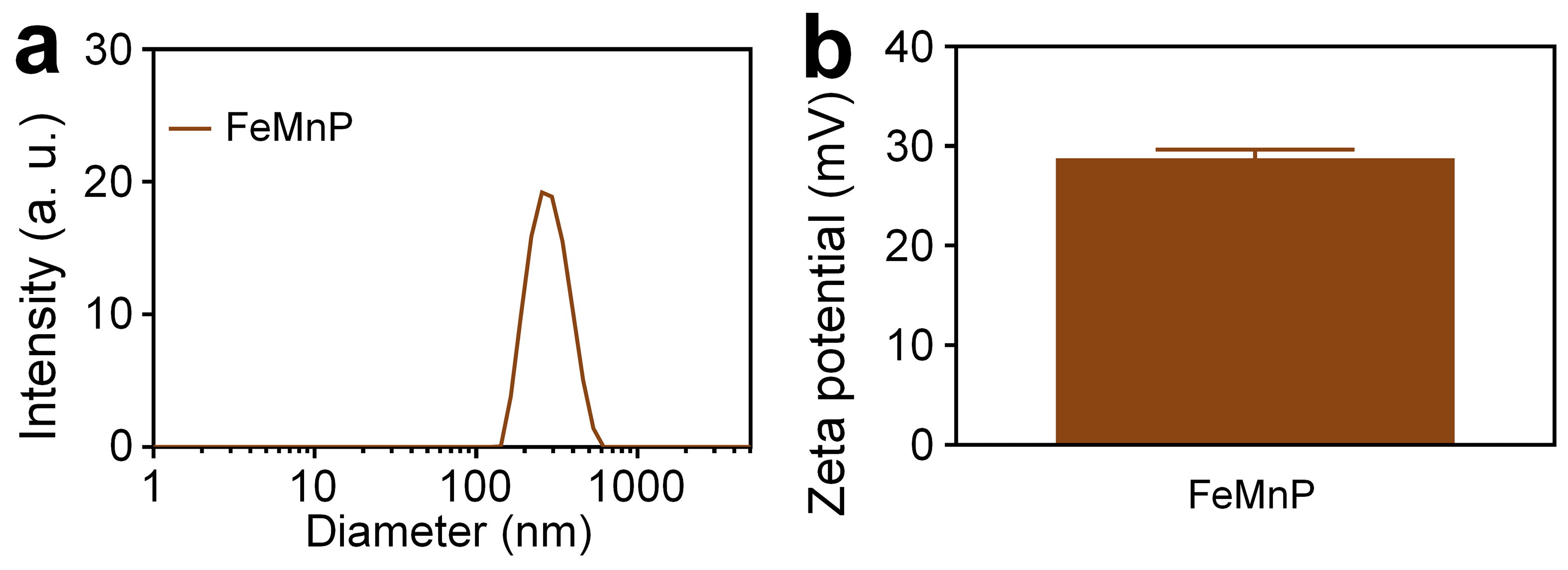


**Figure S9.** (a) Size distribution and (b) zeta potential of FeMnP.


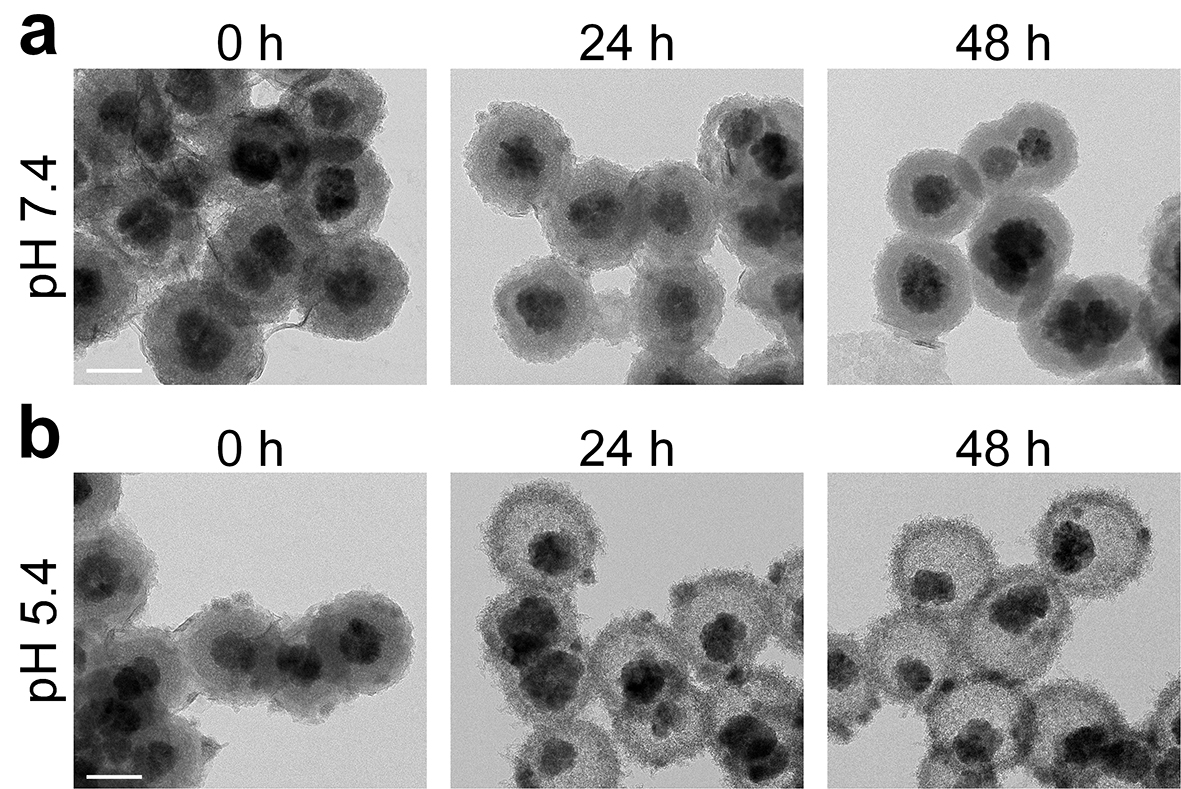


**Figure S10.** The TEM images of FeMnP after incubation in different pH. Scale bars, 100 nm.


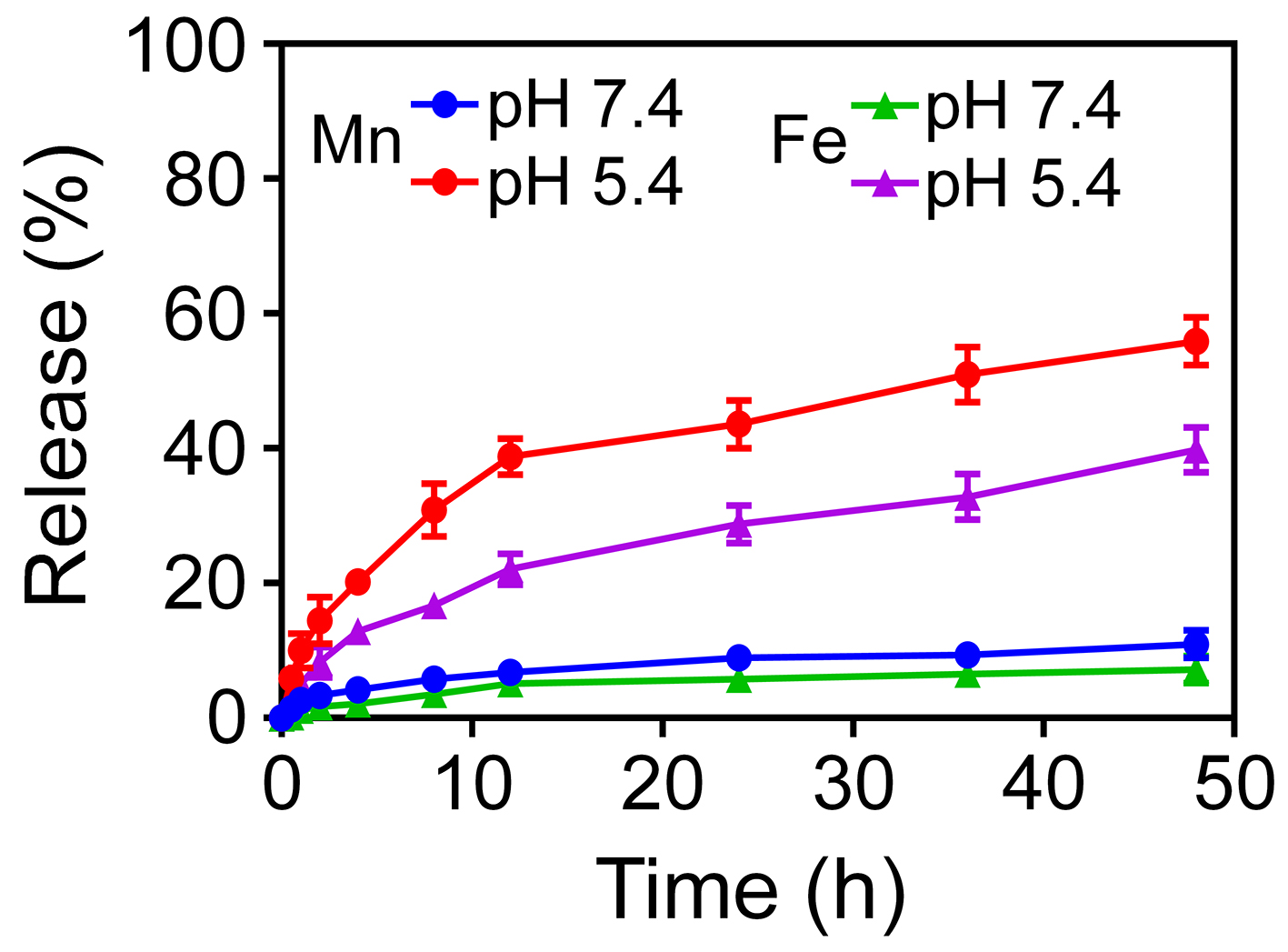


**Figure S11.** Mn and Fe release profiles of FeMnP in PBS with different pH values.


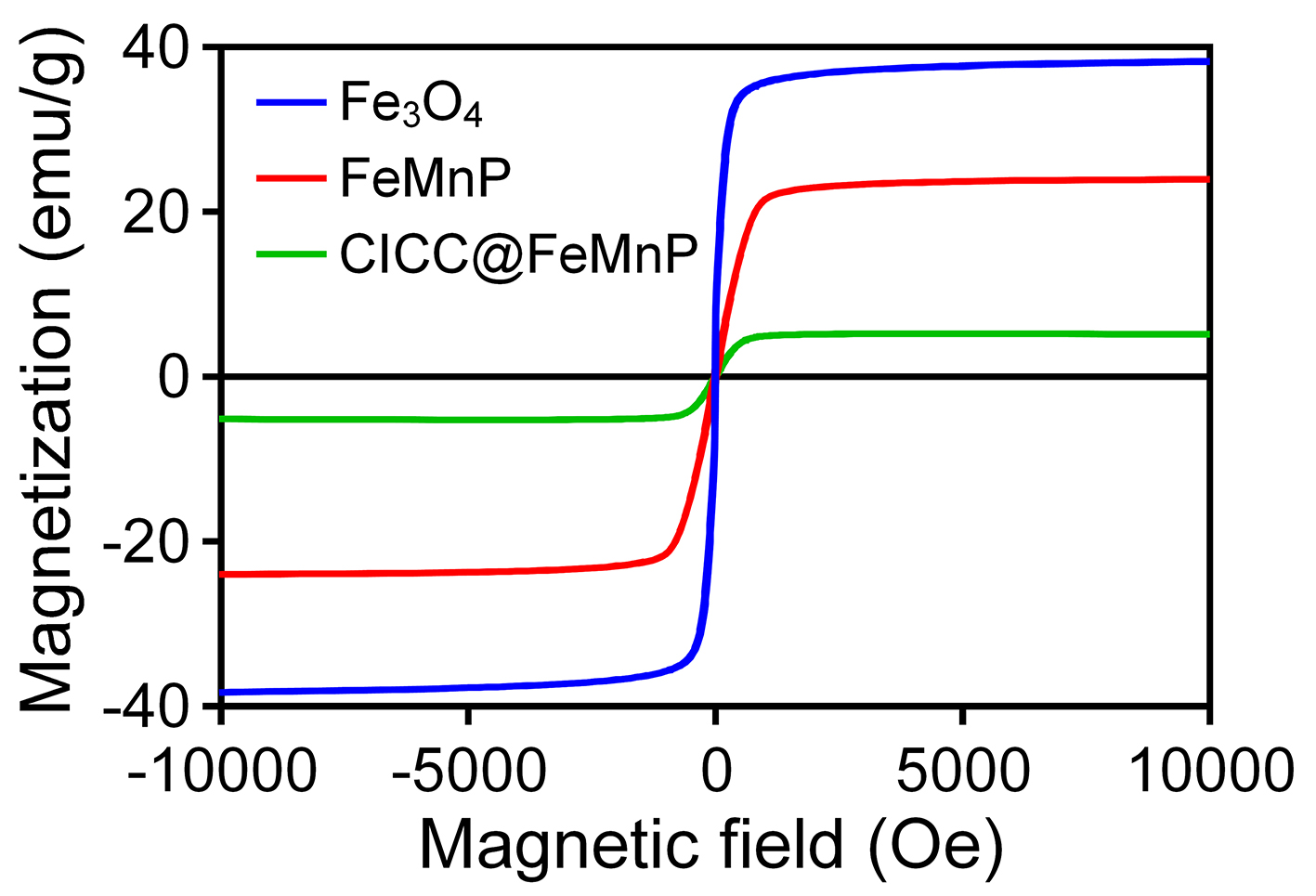


**Figure S12.** The magnetic hysteresis loops of Fe_3_O_4_, FeMnP, and CICC@FeMnP.


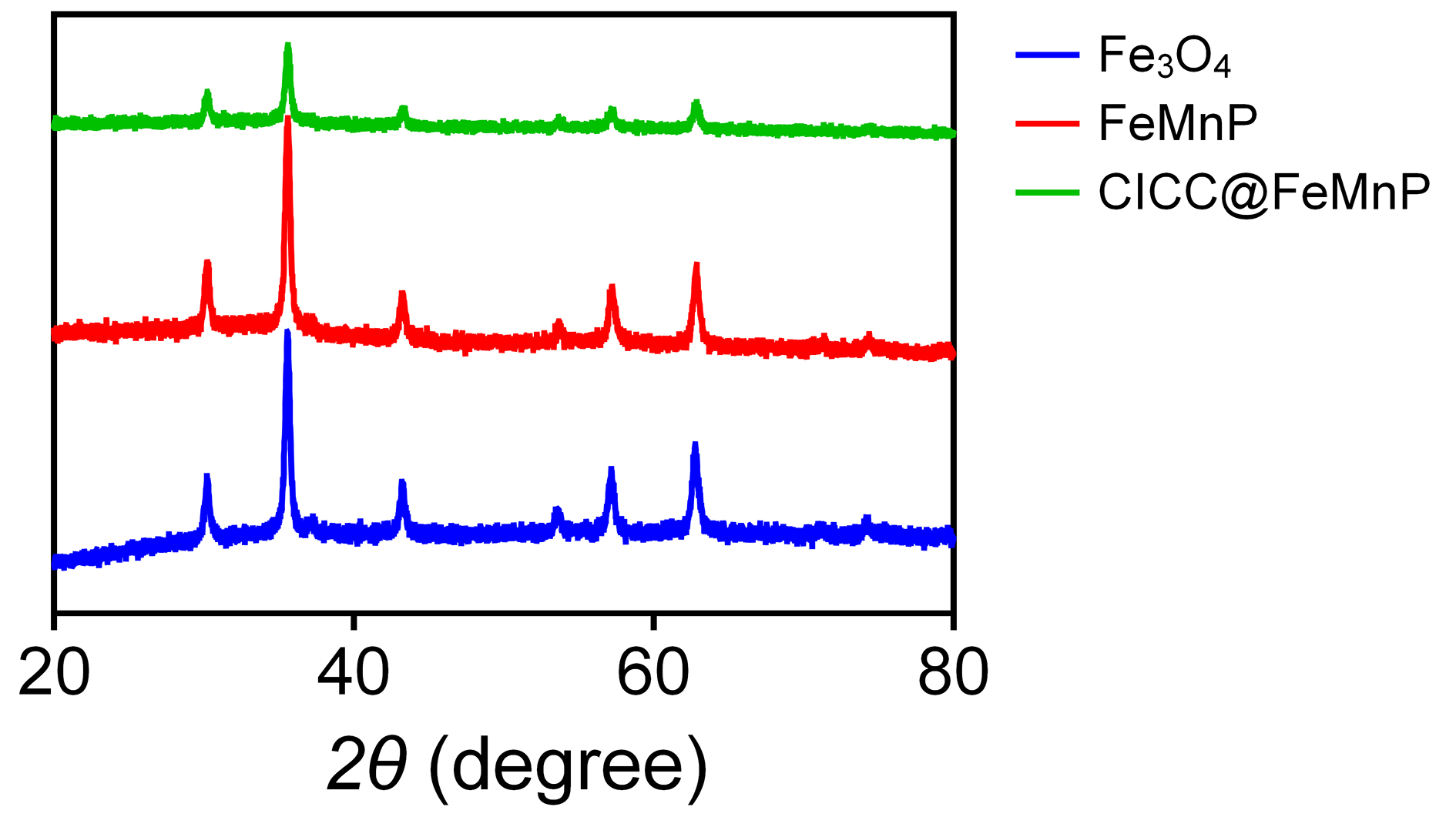


**Figure S13.** XRD patterns of Fe_3_O_4_, FeMnP, and CICC@FeMnP.


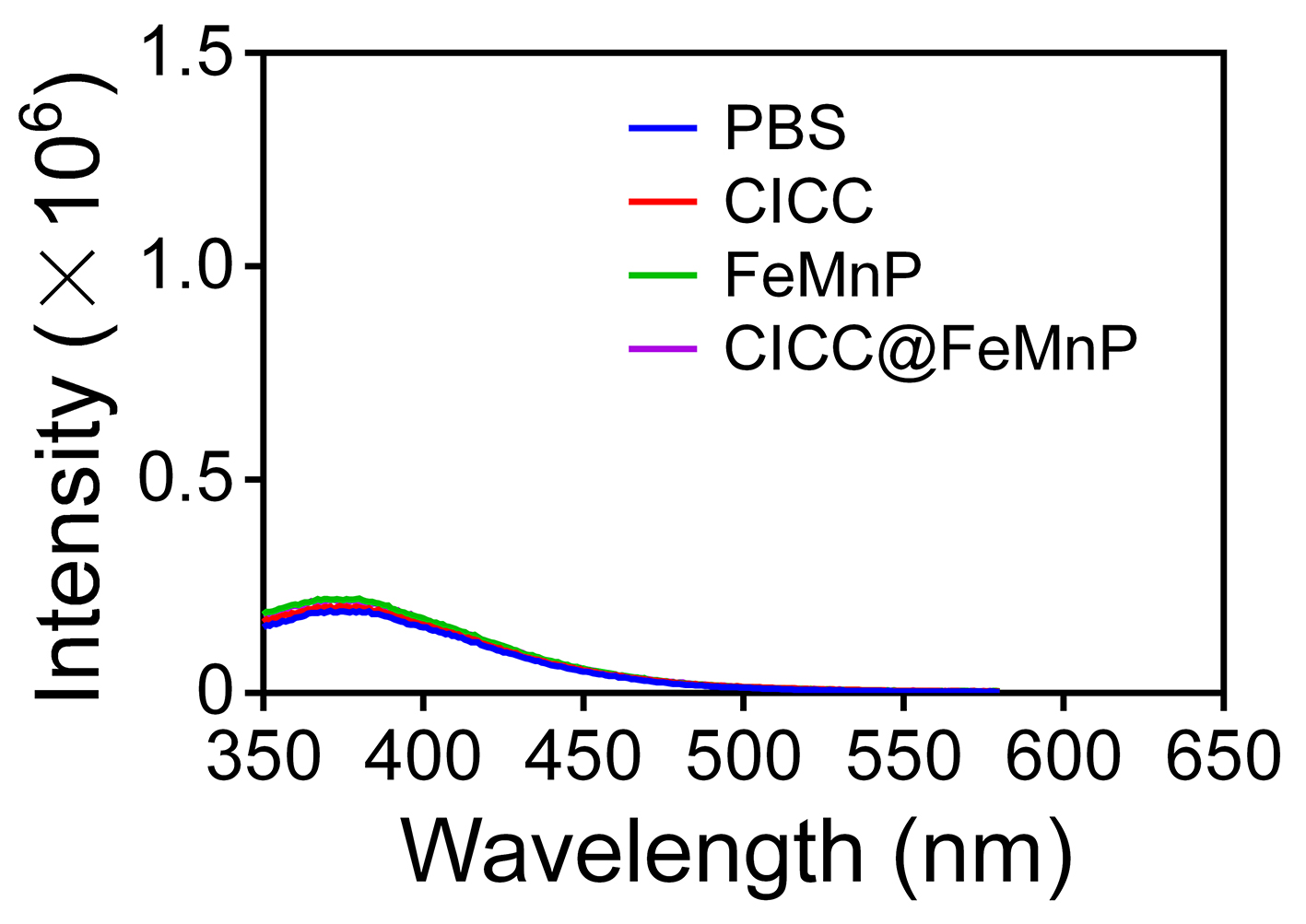


**Figure S14.** Fluorescence spectra of PPTA after different treatments.


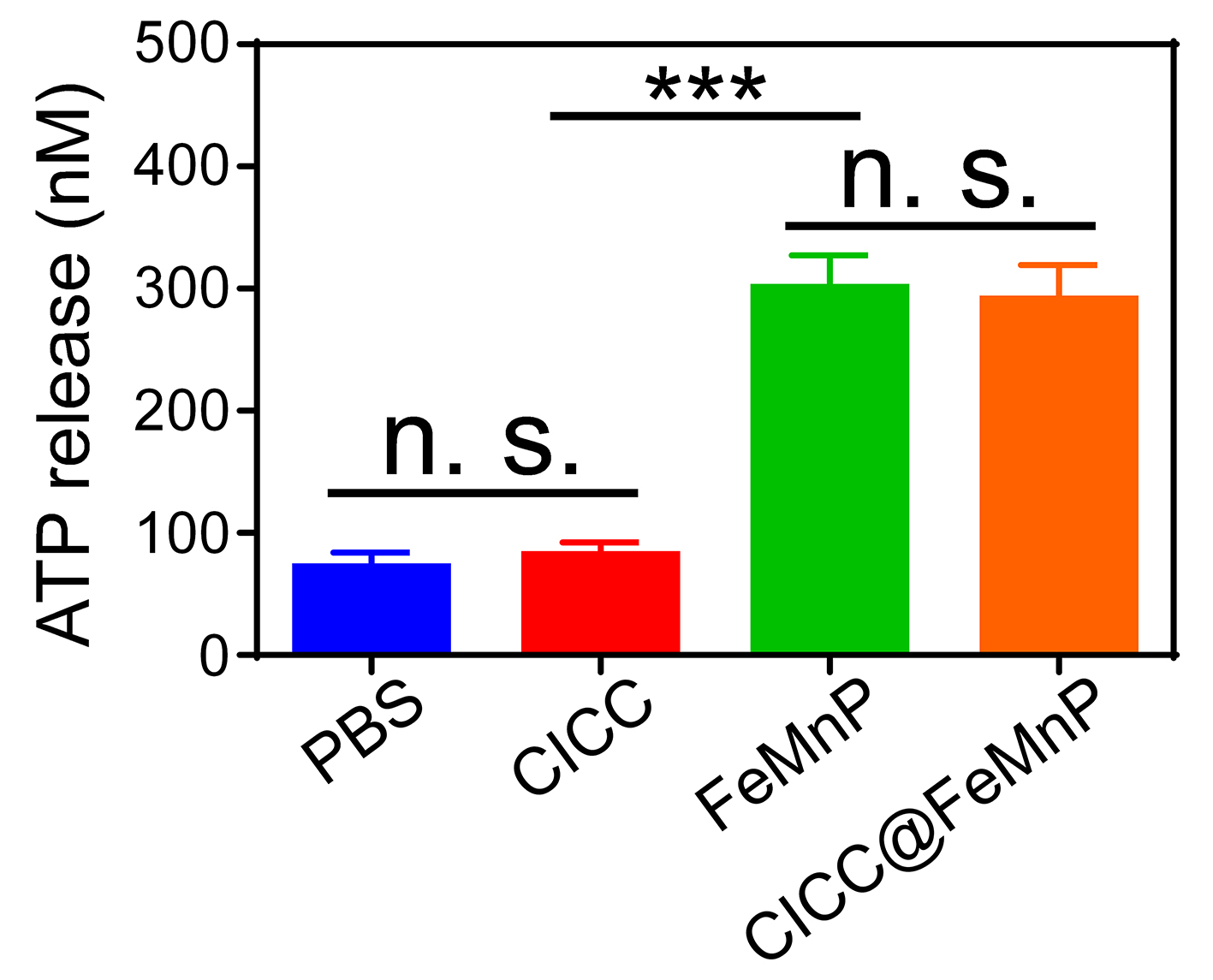


**Figure S15.** Extracellular ATP content after various treatments.


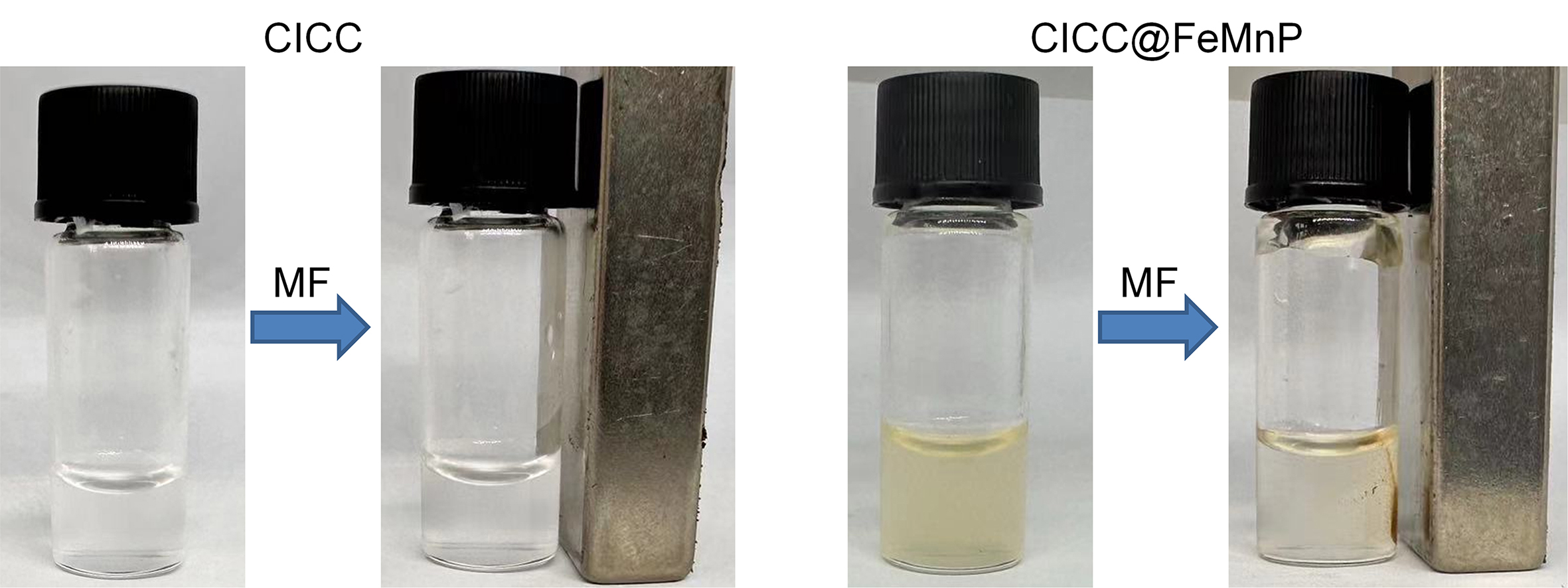


**Figure S16.** Images of CICC and CICC@FeMnP before and after MF action.


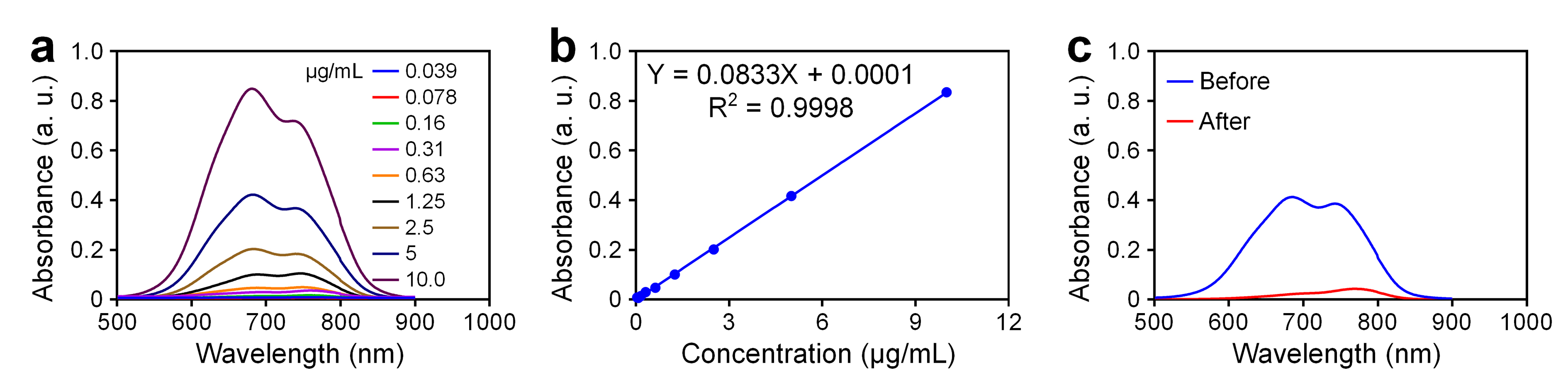


**Figure S17.** (a) UV-vis spectra of different concentrations of DIR. (b) The standard curve of DIR at 690 nm. (c) UV-vis spectra of the DIR before and after incubation with CICC.


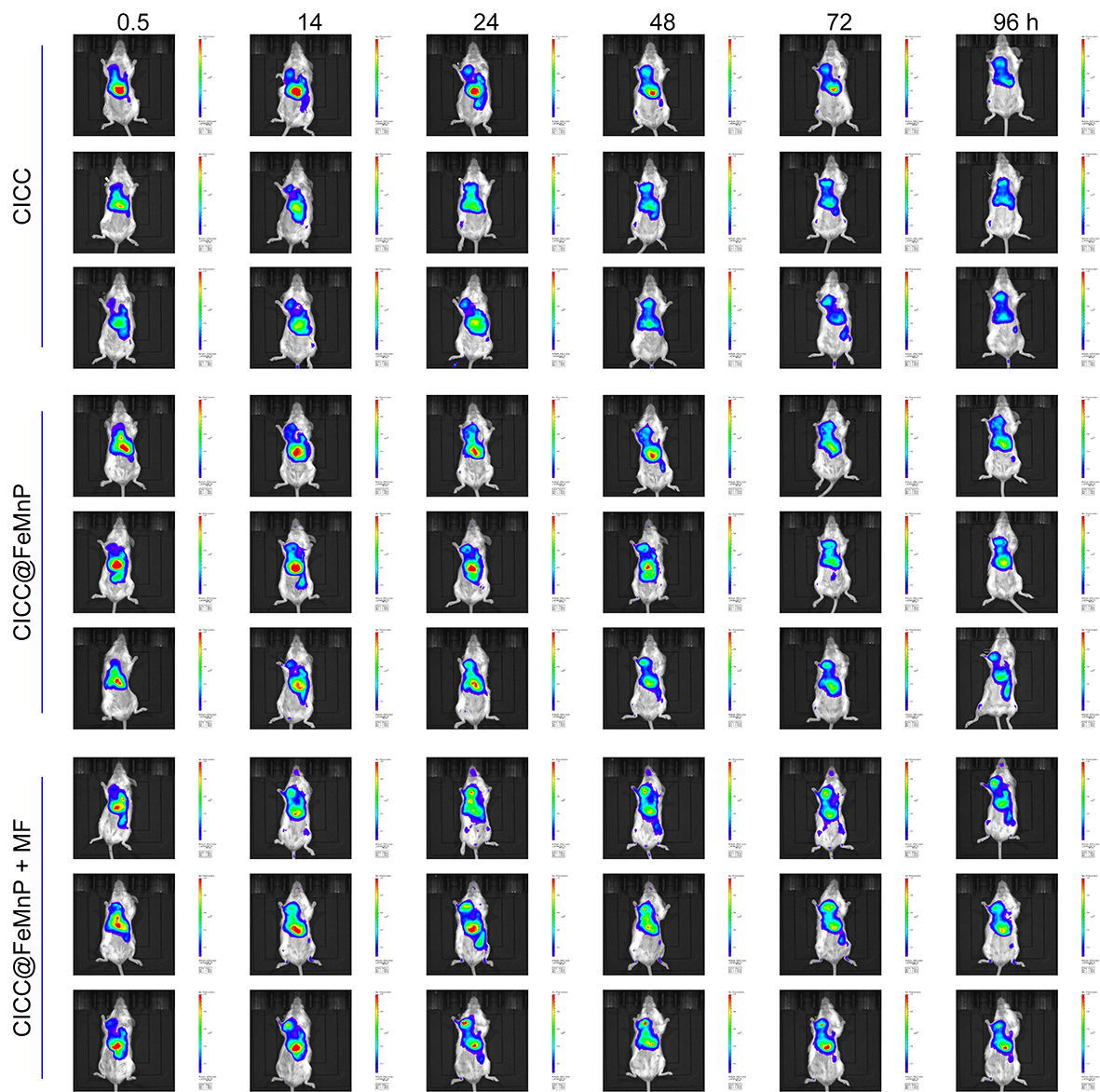


**Figure S18.** In vivo fluorescence images of tumor-bearing mice after intravenous injection of DIR-labeled CICC or CICC@FeMnP, with or without MF.


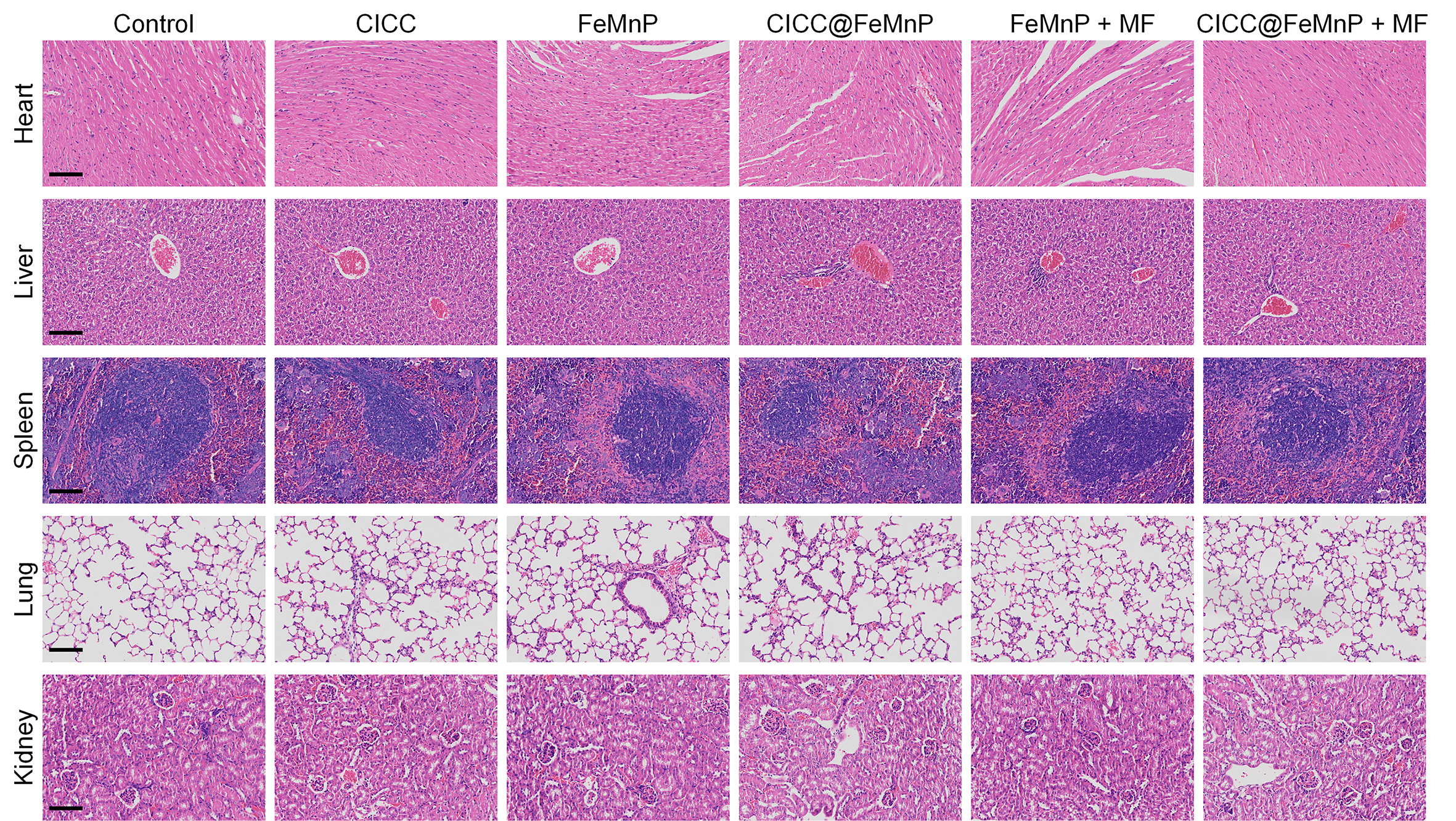


**Figure S19.** H&E staining of major organs. Scale bars, 100 μm.


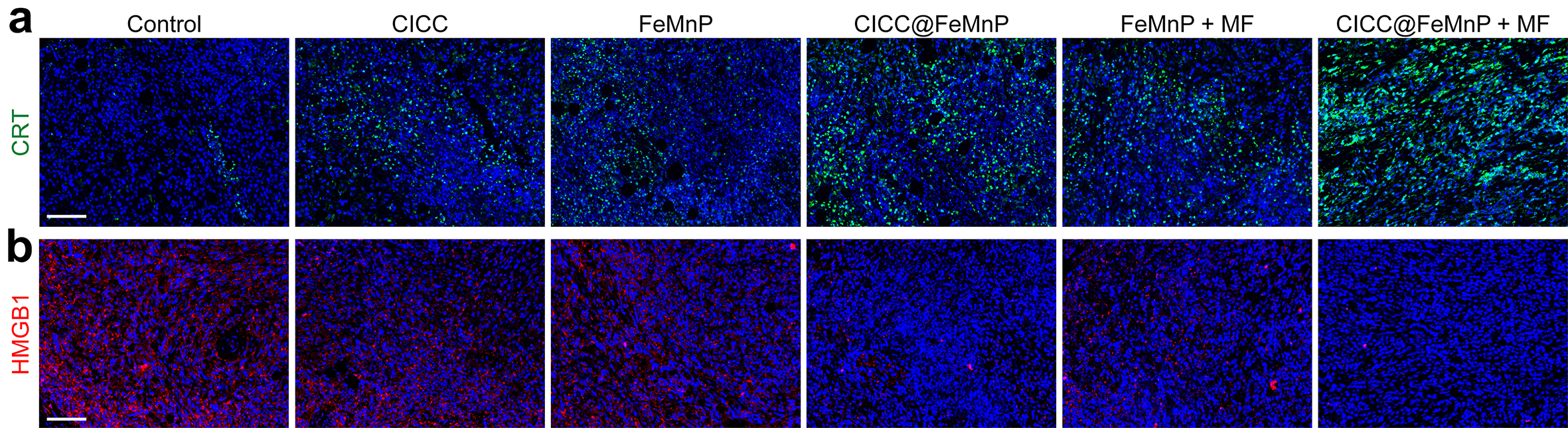


**Figure S20.** Immunofluorescence staining of CRT (a) and HMGB1 (b) in tumor tissues. Scale bars, 100 μm.


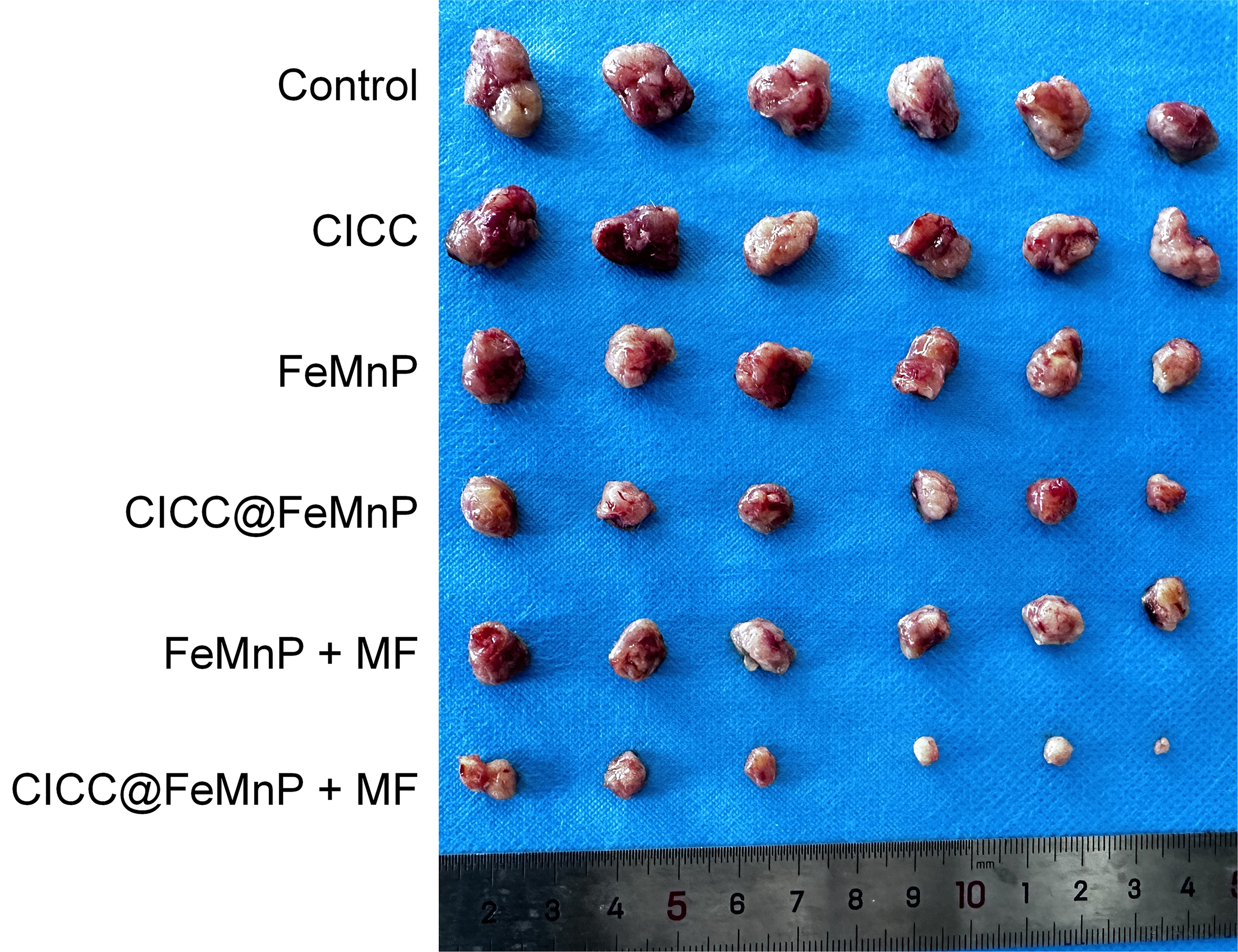


**Figure S21.** Image of recurrent tumors after different treatments.

**Movie S1.** DIL-labeled CICC were incubated in a 6-well plate, and after 15 s, the magnetic field was applied on the right side of the well.

**Movie S2.** DIL-labeled CICC@FeMnP were incubated in a 6-well plate, and after 15 s, the magnetic field was applied on the right side of the well.
